# Learning‐based cognitive control in ADHD: a multicentric study

**DOI:** 10.1111/jcpp.70162

**Published:** 2026-05-05

**Authors:** Lisa Toffoli, Giulia Stefanelli, Fiorella Del Popolo Cristaldi, Gian Marco Duma, Massimiliano Pastore, Vincenza Tarantino, Malida Franzoi, Marilena Vecchi, Alberto Danieli, Federica Martinez, Agnes Blaye, Corentin Gonthier, CALM‐1 Team, Giovanni Mento

**Affiliations:** ^1^ Department of General Psychology – DPG University of Padova Padova Italy; ^2^ Scientific Institute IRCCS E. Medea Conegliano (TV) Italy; ^3^ Department of Developmental Psychology and Socialisation ‐ DPSS University of Padova Padova Italy; ^4^ Department of Psychology, Educational Sciences and Human Movement University of Palermo Palermo Italy; ^5^ Centre de Recherche en Psychologie et Neuroscience (UMR 7077) Aix‐Marseille Université & CNRS Marseille France; ^6^ Nantes Université, Univ Angers, Laboratoire de psychologie des Pays de la Loire, LPPL, UR 4638 Nantes France; ^7^ Institut Universitaire de France Paris France

**Keywords:** Learning‐based cognitive control, ADHD, Flanker task, go‐noGo, inhibition, development

## Abstract

**Background:**

Learning‐based cognitive control (CC), the ability to adapt control strategies based on contextual regularities, has been studied in typically developing (TD) children but remains underexplored in children with attention deficit hyperactivity disorder (ADHD). This study investigates whether children with ADHD show intact learning‐based CC and how it is affected by increased cognitive demands.

**Methods:**

In a multicentric study, 7–14 years old children (145 ADHD [20F, mean = 10.4 ± 2.0], 97 TD [51F, mean = 10.4 ± 2.0]) completed two experimental tasks: a modified Flanker task and a cued go‐noGo task. Both included a List‐Wide Proportion Congruency (LWPC) manipulation to create different contextual predictability (mostly congruent/valid vs. half congruent/valid blocks). Reaction times (RTs) and accuracy were analyzed across blocks, and we also examined associations between task adaptation and neuropsychological (NPS) as well as questionnaire‐based measures of CC, including inhibition, cognitive flexibility, working memory, and behavioral difficulties.

**Results:**

Children with ADHD showed similar LWPC effects to TD peers in the Flanker task, suggesting preserved learning‐based CC in low‐demand contexts. In the cued go‐noGo task, which involved greater attentional and inhibitory demands, children with ADHD displayed less efficient modulation of RTs and reduced speed‐accuracy trade‐off adaptation compared to TD peers. A composite NPS score predicted task adaptation. No associations were found with parental questionnaires.

**Conclusions:**

Learning‐based CC appears preserved in children with ADHD in simple contexts but is hindered under complex, multi‐demand conditions. Neuropsychological performance may be a key mechanism underlying these group differences. These findings emphasize the need for ecologically valid paradigms and tailored interventions targeting complex CC challenges in ADHD.

## Introduction

Attention deficit hyperactivity disorder (ADHD) is a neurodevelopmental condition characterized by disattention and/or hyperactivity and impulsivity (APA, 2013) as well as heterogeneous profiles of cognitive control (CC; Castellanos, Sonuga‐Barke, Milham, & Tannock, 2006; Sonuga‐Barke & Sergeant, 2005; Willcutt, Doyle, Nigg, Faraone, & Pennington, 2005). CC refers to the regulation of thoughts and behavior in the service of goal‐directed actions, supported by executive functions (EFs) such as inhibition, working memory updating, and set shifting (Diamond, 2013, 2020; Miyake & Friedman, 2012). Alterations in these processes are thought to contribute to everyday functional difficulties in ADHD.

Although CC has traditionally been described as a voluntary, top‐down system (Baddeley & Hitch, 1974; Norman & Shallice, 1986), accumulating evidence shows that it is also shaped by bottom‐up learning mechanisms. Performance can adapt automatically based on recent experience and environmental regularities. Classic evidence comes from conflict adaptation effects (Gratton, Coles, & Donchin, 1992), demonstrating trial‐to‐trial adjustments after interference. Beyond these sequential effects, CC can also be modulated by implicit learning of contextual regularities, whereby individuals learn which contexts are more likely to require control and adjust their behavior accordingly (Abrahamse, Braem, Notebaert, & Verguts, 2016; Braem & Egner, 2018; Egner, 2014). A well‐established way to study this learning‐based regulation of CC is through proportion congruency (PC) manipulations, in which the frequency of conflict is varied across blocks of trials (e.g., mostly congruent vs. mostly incongruent contexts). These manipulations induce gradual, experience‐driven adjustments of control: when conflict is more likely, individuals typically engage stronger control and show reduced interference (Bugg & Crump, 2012). PC effects have been observed in adults and in typically developing (TD) children from early childhood (Gonthier, Ambrosi, & Blaye, 2021; Gonthier & Blaye, 2021), suggesting that children can use implicitly learned contextual information to regulate control.

These effects are often interpreted within frameworks proposing that control operates through both reactive and proactive modes (Dual Mechanisms of Control; Braver, 2012). Reactive control is recruited transiently in response to interference, whereas proactive control involves the anticipatory maintenance of goal‐relevant information when conflict is expected. Contextual regularities, such as those induced by PC manipulations, are thought to bias the balance between these modes: more proactive engagement when conflict is frequent, and more reactive responding when it is rare (Chiu & Egner, 2019; Gonthier et al., 2021).

Importantly, both CC and implicit learning have been reported to be atypical in ADHD. Studies suggest differences in the use of contextual information (Luman, Tripp, & Scheres, 2010; Nigg, 2017) and in forms of implicit learning (Barnes, Howard, Howard, Kenealy, & Vaidya, 2010; Huang‐Pollock, Maddox, & Tam, 2014; Laasonen et al., 2014), raising the possibility that children with ADHD may show altered learning‐based regulation of control. Research examining proactive and reactive control in ADHD has yielded mixed findings, with reports of deficits in proactive control, reactive control, or both (Cai et al., 2023; Sidlauskaite, Dhar, Sonuga‐Barke, & Wiersema, 2020; van Hulst et al., 2018). However, no previous studies have directly tested whether children with ADHD use implicitly learned contextual regularities to adapt their level of control.

The present multicentric study (‘CALM‐1’; see Figure S1, Appendix S1) addressed this gap by comparing learning‐based CC in children with ADHD and TD peers (7–14 years). To operationalize learning‐based control, we used list‐wide PC manipulations in two tasks that place different demands on control processes.

First, a modified Flanker task (Gonthier et al., 2021) required children to respond to a central arrow while ignoring congruent or incongruent flankers. Here, PC manipulations varied the frequency of interference across blocks, allowing us to assess learning‐based modulation of interference control. Second, a cued go/noGo task (‘Addy task’) required children to prepare a response based on a cue and withhold responses on noGo trials. PC manipulations varied the predictability of valid versus invalid cue‐target relations, enabling us to test learning‐based adjustments of attentional preparation and motor inhibition under higher cognitive demands.

Using both tasks allowed us to examine whether PC‐driven adaptation differed between groups and across control domains, and whether effects were modulated by increasing task complexity.

Based on previous literature, we expected TD children to use implicit regularities to regulate CC in both tasks, irrespective of age. Specifically, a PC effect was expected in the Flanker task (i.e., larger congruency effects in the half‐congruent than in the mostly congruent block; Gonthier et al., 2021) and a modulation of the validity effect in the Addy task (i.e., larger validity effects in the half‐valid than in the mostly valid (MV) block; Mento et al., 2022). In other words, the RT cost for incongruent/invalid relative to congruent/valid trials was expected to be smaller in the half‐congruent (50–50) than in the mostly congruent condition, without a loss in accuracy.

Although these effects were expected across all ages, we anticipated possible developmental improvements in learning‐based CC, as older children may be better at detecting regularities or implementing control. The direction of such developmental change was left open, given mixed evidence in the literature.

For the ADHD group, since PC manipulations have not been previously examined, we formulated exploratory hypotheses. Based on evidence of atypical implicit learning and CC in ADHD, possible outcomes included reduced or absent PC effects, or preserved effects only in the Flanker task compared to the Addy task, as the latter implies multiple cognitive demands.

## Methods

### Ethics statement

All parents provided written consent, and children provided oral assent after procedure explanation, following Italian ethical guidelines for research (Associazione Italiana di Psicologia, 2022). All experimental procedures were approved by the Ethics Committee (School of Psychology—Padova University, protocol no. 4920 of August 8, 2022) and followed the principles expressed in the Declaration of Helsinki.

### Participants

The initial cohort included 303 children and adolescents aged 5–14 (154 ADHD, 149 TD). The number of participants was not determined through a formal power analysis but rather based on a feasibility evaluation of our resources (Lakens, 2022). Inclusion in the ADHD group was based on a preexisting formal diagnosis, according to DSM‐5 criteria with ICD‐10 code assignment, as established by a clinical psychologist or neuropsychiatrist. The TD group included only children with no reported formal diagnosis referring to neurodevelopmental or neurological/psychiatric conditions, as assessed through a parent‐report screening survey (see Appendix S2) that evaluated developmental and medical diagnoses, medication use, family history of developmental disorders and perinatal history. All participants had normal or corrected vision.

Both the Flanker and Addy task samples were drawn from this same initial cohort. However, final sample sizes differed across tasks due to the application of exclusion criteria (see Table S1, Appendix S2). The final Flanker sample included 132 children with ADHD (16 females, mean age 9.8 ± 2.0) and 83 TD children (41 females, mean age 9.7 ± 2.0). The Addy task had 122 children with ADHD (19 females, mean age 9.9 ± 1.9) and 91 TD children (48 females, mean age 9.8 ± 2.0) (see Figure S2, Appendix S1). The higher male prevalence in ADHD reflects typical gender ratios and was controlled for in analyses (Davidovitch, Koren, Fund, Shrem, & Porath, 2017; G. Polanczyk, De Lima, Horta, Biederman, & Rohde, 2007; Xu, Strathearn, Liu, Yang, & Bao, 2018).

### Materials

#### Flanker task

##### Trial structure

After an initial fixation cross (850 ms), a visual stimulus appeared and remained on screen until participant's response or for a maximum of 3,000 ms. The stimulus depicted five arrows pointing either left, right, up or down. In congruent trials, the target arrow (i.e., central arrow) pointed toward the same direction as the flanker arrows (i.e., lateral arrows; e.g., <<<<<), whereas in incongruent trials, the target and the flankers pointed toward different directions (e.g., <<> < <). Instructions prompted participants to press—as fast and accurately as possible—a button (left or right) based on the target direction while ignoring the distracting flankers. The trial list was randomized.

##### List‐wide proportion congruency

**Figure 1:**
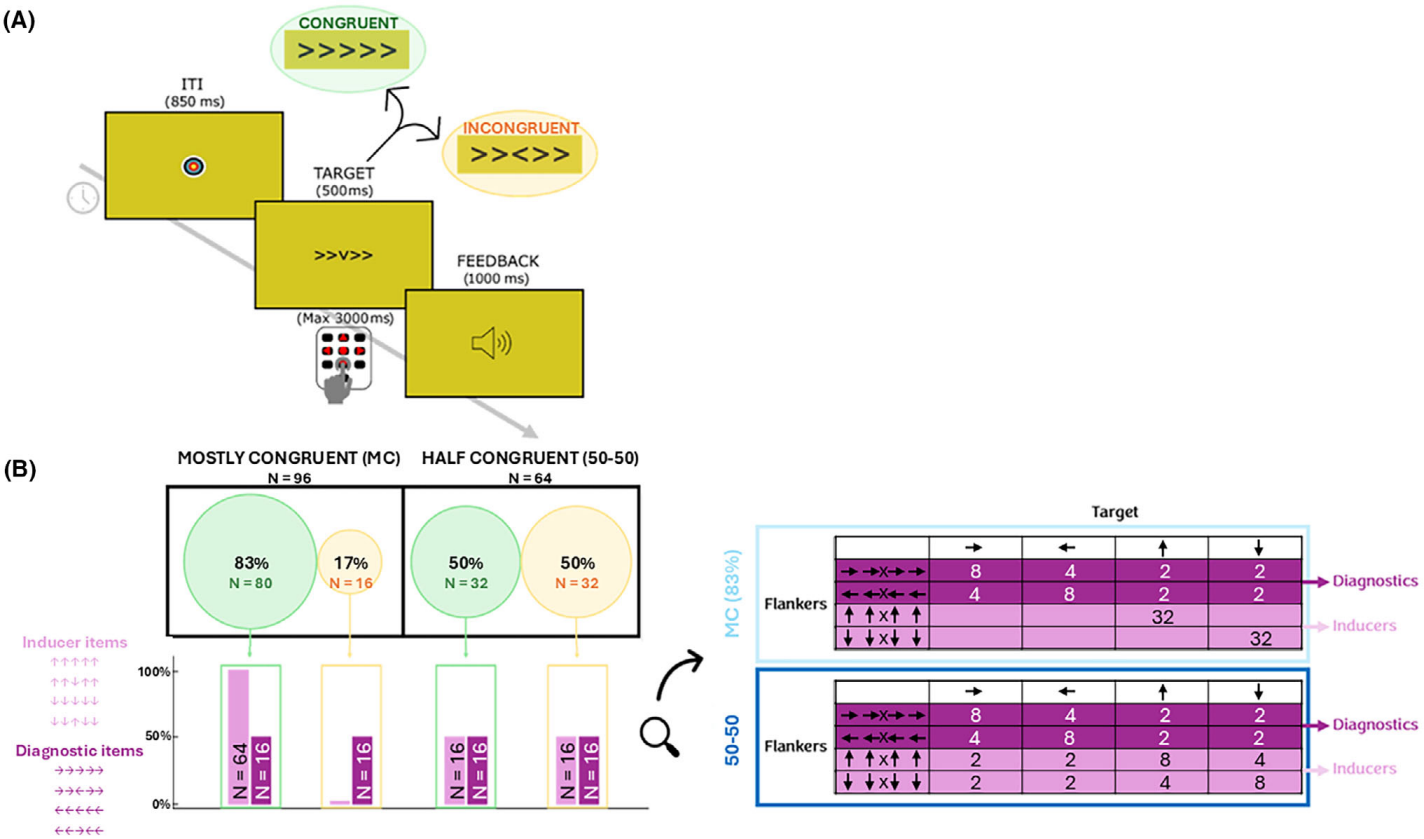
Flanker task. Panel A. After a fixation intertrial interval (ITI) (850 ms), the target stimulus appears on the center of the screen. The target consisted in a string of a central arrow pointing left or right and lateral arrows (i.e., flankers) pointing in the same (i.e., congruent) or opposite (i.e., incongruent) direction. Panel B. A list‐wide proportion congruency manipulation was used to create a mostly congruent (MC, 83% congruent trials) and a half congruent (50–50, 50% congruent trials) block. Inducer items (pink) were 100% congruent in the MCblock, and 50% congruent in the 50–50 block. Conversely, diagnostic items (violet) were 50% congruent in both blocks. In order to motivate children, the following oral and written instructions were given: ‘Would you like to help Maty become an archery champion? You will see a target sign appear in the center of the screen. Then you will see five arrows, but you need to focus on the central one while ignoring the later ones: that's the correct arrow that Maty needs to use in order to win! If the central arrow points on the left, press left, if the central arrow points on the right, press right’. Children completed a 12‐trial practice with visual (‘GREAT’, ‘OH NO… WRONG ANSWER!’ and ‘FASTER!’) and auditory feedback of 1,000 ms (high tone for correct, low tone for wrong or slow). Three breaks were scheduled after trials 32, 64, and 112 to reduce fatigue and maintain motivation, supported through verbal appraisal. The software E‐prime (version 2.08.90; Schneider, Eschman, & Zuccolotto, 2002) was used to create and administer the Flanker task (Gonthier et al., 2021)

Two trial types were defined by target‐flanker arrow correspondence: congruent (target and flankers point the same way) and incongruent (target and flankers point differently; see Figure 1A). A list‐wide proportion congruency (LWPC) manipulation was used to create a mostly congruent (MC, 83% congruent trials; 96 trials: 80 congruent, 16 incongruent) and a half congruent (50–50, 50% congruent trials; 64 trials: 32 congruent, 32 incongruent) block. This manipulation is intended to reduce the RT cost for incongruent relative to congruent trials when moving from the MC to the 50–50 block, without a loss in accuracy.

To separate implicit learning from adaptive control (Braem et al., 2019; Gonthier et al., 2021), both blocks included inducer and diagnostic trials. Inducers (i.e., flankers pointing up or down) were 100% congruent in the MC block (*N* trials = 64 congruent), and 50% congruent in the 50–50 block (*N* trials = 16 congruent and 16 incongruent); diagnostics (i.e., flankers pointing left or right^1^) were 50% congruent in both the MC (*N* trials = 16 congruent, 16 incongruent) and the 50–50 block (*N* trials = 16 congruent, 16 incongruent), making them suitable to compare the two blocks (see Figure 1B).

The block order was fixed (i.e., MC first, then 50–50), and the task lasted about 10 min. During the experiment, trial‐by‐trial auditory feedback was provided during this task to replicate the methodology of Gonthier et al. (2021) and to sustain motivation given the monotonous nature of the task.

#### Addy task

##### Trial structure

After an initial fixation cross (800–1,200 ms), the trial started with a visual cue, which was depicted as an arrow pointing left or right, for 500 ms. A target represented by the cartoon of a male or female astronaut then appeared after an interval time of 1,500 ms and remained on screen until the participant's response or for a maximum of 3,000 ms. Two astronaut icons were continuously displayed on the left and right bottom of the screen. Instructions prompted participants to press—as fast and accurately as possible—a button (left or right) based on the correspondence between the target and the lower astronaut icons (go trials). Finally, when an alien was present on the astronaut's spacesuit, participants had to press no button (noGo trials). The position of the astronaut icons as well as the trial list were counterbalanced between participants.

##### List‐wide proportion congruency

**Figure 2:**
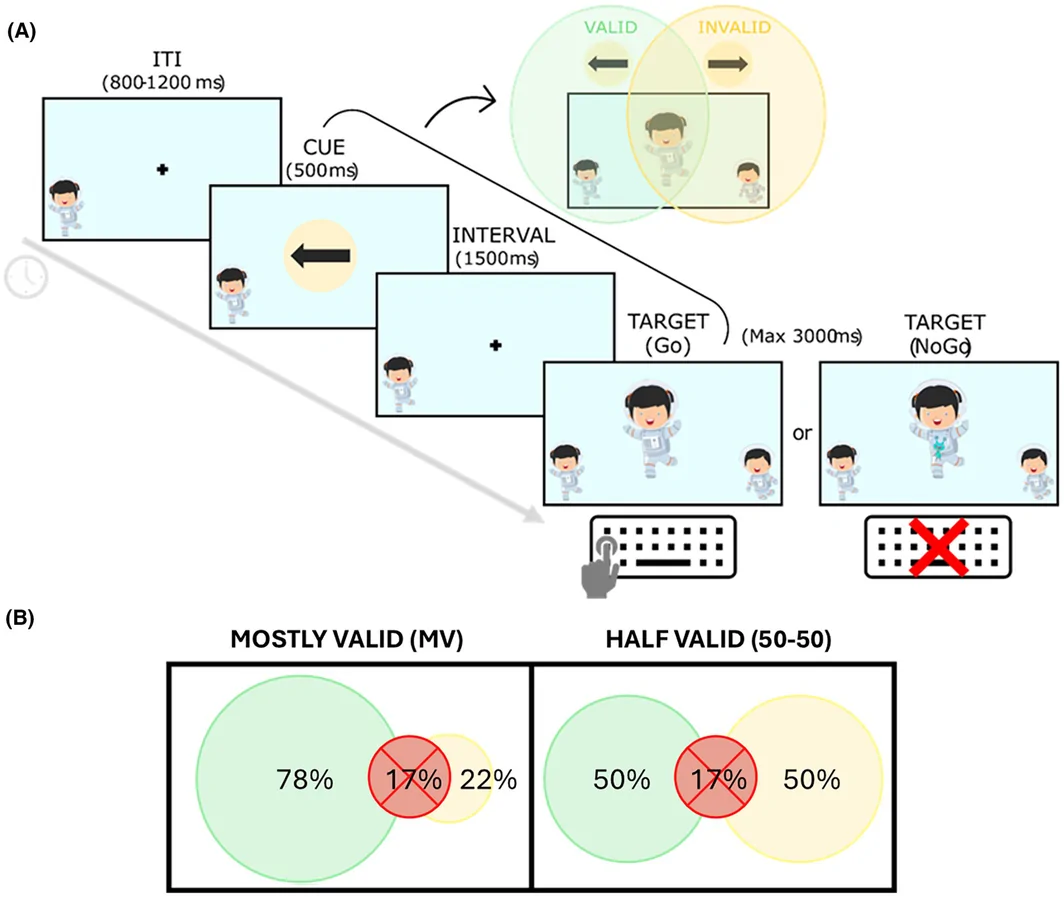
Addy task. Panel A. After a fixation intertrial interval (ITI) (800–1,200 ms), the cue (arrow left or right) appears on the center of the screen (500 ms), followed after 1,500 ms by the target stimulus (male or female astronaut). Participants had to press a button (left or right) based on the correspondence between the target and the icons on the bottom left and right: if it was coherent with the cue direction the trial was valid; otherwise it was invalid. Panel B. A list‐wide proportion congruency manipulation was used to create a mostly valid (78% valid trials) and a half valid (50–50, 50% valid trials) block. Moreover, a fixed percentage of noGo trials (17%) was present in both blocks to assess response inhibition in different predictive contexts. In order to motivate children, the following oral and written instructions were given: ‘Would you like to help astronauts Nick and Maty find these little stars that got lost in space? You will see a space sign appearing in the center of the screen that will help Nick and Maty find the stars. Look at it carefully because the arrow can point either to the right or to the left. Then you will see an astronaut: if you see the female astronaut, press her button, while if you see the male astronaut, press his button. However, if you see an alien, do not press any button’. Children completed a 14‐trial practice with visual feedback (happy face for correct, puzzled face for wrong or slow) to ensure understanding. Four breaks occurred after every 36 trials to reduce fatigue and keep motivation high through verbal appraisal. The software E‐prime (version 2.08.90; Schneider et al., 2002) was used to create and administer the Addy task, which is a cued go‐noGo task

Two trial types were defined by cue‐target correspondence: valid (when the arrow direction correctly anticipated the required response) and invalid (when the arrow direction did not anticipate the required response; see Figure 2A). An LWPC manipulation was used to create an MV (78% valid trials; 72 trials: 56 valid, 16 invalid) and a half‐valid (50–50, 50% valid trials; 72 trials: 36 valid, 36 invalid, 50% validity) block. As for the Flanker task, this manipulation is intended to reduce the RT cost for invalid relative to valid trials when moving from the MC to the 50–50 block, without a loss in accuracy. Moreover, the same fixed percentage of noGo trials (17%) was present in both blocks (12 trials per block, equally valid and invalid) to assess response inhibition as a function of predictive context (see Figure 2B). Block order was fixed (i.e., MV first, then 50–50), and the task lasted about 10 min. During the experiment, no trial‐by‐trial feedback was provided, as the task's cognitively demanding nature was expected to adequately sustain engagement without external reinforcement.

#### Cognitive and neuropsychological measures

Nonverbal reasoning was assessed using the *colored progressive matrices* (CPM, Raven, 1990). Inhibitory control and cognitive flexibility were measured using several standardized neuropsychological (NPS) tasks, including the *Numerical Stroop* (BIA‐R, Marzocchi, Re, & Cornoldi, 2010), the *NEPSY‐II Inhibition* subtest (Korkman, Kirk, & Kemp, 2007), and the Wisconsin Card Sorting Test (WCST, Grant & Berg, 1948; Heaton, 1981) administered in the 64‐card computerized version, using the PEBL software (version 2.1; Mueller & Piper, 2014). Task descriptions and descriptive statistics are provided in Table S2, Appendix S2.

#### Parental questionnaires

To assess children's everyday behavior and executive functioning, parents completed the Conners' Parent Rating Scale‐Revised (CPRS‐R, Conners, Sitarenios, Parker, & Epstein, 1998; Nobile, Alberti, & Zuddas, 2007), the Child Behavior Checklist (CBCL, Achenbach & Edelbrock, 1991), the Behavior Rating Inventory of Executive Function‐2nd Edition (BRIEF‐2, Gioia, Isquith, Guy, & Kenworthy, 2015), and the Intolerance of Uncertainty Scale‐Parent Version (IUS‐P, Bottesi, Noventa, Freeston, & Ghisi, 2019; Comer et al., 2009). Questionnaires' descriptions and descriptive statistics are provided in Table S3, Appendix S2.

### Experimental procedure

This study took place in the context of a large Italian multicenter project led by the NeuroDev lab at the University of Padova, in collaboration with 10 clinical centers across Veneto, Liguria, and Emilia‐Romagna (the CALM‐1 team). Families of children with ADHD were recruited through this network: Families received study information from their clinicians, and if interested, provided written informed consent for participation. TD participants were recruited from kindergarten, primary, and secondary schools in the Marche (AN) (‘Maria Montessori’ and ‘Raffaello Sanzio’) and Sicily (PA) (‘Antonio Gramsci’ and ‘Giovanni Falcone’) regions. Teachers presented study information to families, and all children whose parents provided written informed consent were included in the study.

Data were collected between December 2022 and June 2023, involving two experimental tasks (Flanker and Addy) and a nonverbal reasoning test (CPM; Raven, 1990). Participants were tested individually in quiet, well‐lit rooms free from distractions, either at their school or at their clinical center. Throughout the experimental session, a trainee was present in the room with the participant. They were seated approximately 60 cm from a laptop (12.5 inches, resolution of 1,536 × 1,024 pixels). Apart from the CPM, the NPS testing was conducted only for children in the ADHD group. These NPS tests were administered either on the same day of the experimental session or across multiple sessions (typically 2), based on the scheduling of individual visits at the referral clinical center. Each experimental session lasted approximately 60 min, with breaks included to prevent participant fatigue. At the end of the experimental session, participants received a *Young Scientist Certificate* as thanks for their contribution. No other forms of compensation were provided.

Parents of both TD children and children with ADHD completed questionnaires using the online platform Qualtrics (Qualtrics, 2019), providing additional insights into their children's functioning.

### Statistical analysis

The analyses were conducted using generalized linear mixed models (GLMMs) on both RTs and accuracy. Reaction times (RT, expressed in seconds) were analyzed after removing premature (i.e., <150 ms before target onset), delayed (i.e., >2,500 ms after target onset), and incorrect (i.e., omission or commission errors) responses. Regarding accuracy, we analyzed all responses provided between 150 ms and 2,500 ms (also keeping noGo trials with no response, RT = 0 ms). In the case of the flanker task, only diagnostic trials were analyzed.

For the Flanker task, the study had a 2 (within‐subject, *trialType*: congruent, incongruent) × 2 (within‐subject, *block*: MC, 50–50) × 2 (between‐subject, *group*: ADHD, TD) design. For the Addy task, the study had a 3 (within‐subject, *trialType*: go valid, noGo valid, go invalid) × 2 (within‐subject, *block*: MV, 50–50) × 2 (between‐subject, *group*: ADHD, TD) design. For both RTs and accuracy, the GLMM models (see Table 1) included a random intercept and by‐participant random slopes for *trialType*, *block*, and their interaction.

**Table 1:** Fitted models

Fitted models	
Mod_commissions_Flanker & Mod_commissions_Addy	*Dependent variable* = accuracy (correct responses and commissions) *Fixed effects* = 1 + Block × TrialType × Group × Age + Sex *Random intercept and by‐participant random slopes* = 1 + Block × TrialType *Family distribution* = binomial with logit link function
Mod_RT_Flanker & Mod_RT_Addy	*Dependent variable* = RT (seconds) *Fixed effects* = 1 + Block × TrialType + Block × TrialType × Group × AGE + preRT + Acc(*n*−1) *Random intercept and by‐participant random slopes* = 1 + Block × TrialType *Family distribution* = Inverse gaussian with logarithmic link function
Mod_quest_Addy	*Dependent variable* = delta RT valid trials & delta RT invalid trials *Fixed effects* = questionnaires' composite score + Group + Age *Family distribution* = Gaussian with identity link function
Mod_NPS_Addy	*Dependent variable* = delta RT valid trials & delta RT invalid trials *Fixed effects* = NPS' composite score + Age *Family distribution* = Gaussian with identity link function

Fitted models for accuracy (Mod_commissions), reaction times (Mod_RT) and for the association between performance adaptation and questionnaires/neuropsychological tests (Mod_quest_Addy & Mod_NPS_Addy).

In both cases, *Age* (in months) was included as an interaction factor to examine potential developmental changes in PC effects across the groups. Instead, *Sex* was included in all analyses as a subject‐level covariate to control for baseline differences, as it was not expected to influence PC effects. To deal with potential trial‐by‐trial sequential or fatigue effects, the model included the following covariates: (1) the RT in the preceding trial (*preRT*, continuous numeric variable, scaled) to account for the temporal dependency in response times (Baayen & Milin, 2010); (2) the accuracy on the preceding trial (*Acc(n − 1)*; 0 = incorrect, 1 = correct) to account for posterror slowing (Rabbitt, 1966); and (3) the type of trial on the preceding trial (*Trial(n −* 1); 0 = congruent/valid, 1 = incongruent/invalid or noGo) to account for sequential effects (Horga et al., 2011).

In addition, as part of an exploratory analysis, linear models (LMs) assessed associations between task performance and questionnaires/NPS scores. Importantly, these models were fitted only when the task analyses showed group differences in CC adaptation. This approach was chosen to specifically examine whether the processes differentiating the groups in terms of learning‐based CC were related to broader behavioral and NPS functioning and to reduce the risk of inflating Type I error due to multiple comparisons.

All statistical analyses were performed in R (R Core Team, 2020; version 4.3.2) using a Bayesian framework with the ‘brms’ package (Bürkner, 2017). Weak informative and zero‐centered priors were used. Full model details (chains, samples, burn‐in, family, and priors) can be found in the Supporting Information (see Tables S4–S9, Appendix S3). Results' tables report estimated parameters with 95% highest density intervals (HDI) [lower, upper]. The HDI is the posterior distribution that includes the most credible values of the true parameter (Makowski, Ben‐Shachar, & Lüdecke, 2019). Hypothesis testing used probability of direction (PD) from the ‘bayestestR’ package (Makowski et al., 2019), indicating effect direction certainty (0.5–1 scale, with values near 1 implying strong directional evidence), correlated with frequentist *p*‐values. PD values equal to or greater than 97.5% were considered to indicate a probable effect (Makowski et al., 2019). Post hoc pairwise comparisons were done using estimated marginal means contrasts (‘emmeans’ package, Lenth, 2020), reporting estimates (b), standard errors (SE), and standardized beta coefficients.

All analyses were also run using frequentist methods, with those results provided in Tables S10–S13, Appendix S4. Importantly, the main findings were consistent across the two analytical approaches.

#### Predicting adaptation from cognitive/NPS measures and questionnaires

Further exploratory analyses examining the association between cognitive and NPS measures and experimental tasks were conducted exclusively for tasks that showed a group effect in adaptation. The Addy task was the only one that met this criterion (see Results section). These analyses were performed on a subsample, as not all participants completed the questionnaires (*N* = 90 ADHD [10.6 ± 1.9, 13F], 30 TD [9.5 ± 2.0, 16F]), and only the ADHD group underwent the NPS tests (*N* = 98 ADHD [10.4 ± 1.9, 16F]). Descriptive statistics are reported in Appendix S2, Tables S2 and S3. To reduce the risk of inflating Type I error due to multiple comparisons, we computed composite scores for both the questionnaires and the NPS tests. These composites were intended to summarize related measures that tap into common underlying constructs, as supported by acceptable average intercorrelations among the included variables (*r* = .61 for questionnaires; *r* = .25 for NPS tests). The questionnaire composite included the total scores from the CPRS Global Index, CPRS DSM‐IV total, BRIEF‐2 Global Executive Composite (GEC), CBCL Total Problems, and IUS‐P. The NPS composite included the main performance outcomes from the Numerical Stroop (errors and reaction time), WCST (perseverative errors and perseverative responses), and Nepsy Inhibition (errors and reaction time) tests. Before aggregation, each variable was standardized (*z*‐scores) to place them on a common scale. Variables were reverse‐scored when necessary so that higher values consistently indicated better performance. The composite score for each domain (questionnaires and NPS tests) was then computed as the mean of the standardized scores across the corresponding variables. Internal consistency, as assessed via McDonald's Omega Total (ω_t_), indicated acceptable reliability for both the questionnaire (ω_t_ = 0.9) and NPS (ω_t_ = 0.7) composites (Dunn, Baguley, & Brunsden, 2014).

Two separate linear models were fitted: one including the questionnaire composite score and the other including the NPS composite score as predictors. In both models, age was entered as a covariate. The group variable (TD vs ADHD) was included only in the questionnaire model, as both groups had parental questionnaire data. By contrast, only children with ADHD completed the NPS tests; thus, the TD group could not be included in the NPS model.

As dependent variables, we calculated difference (delta) scores between blocks. These difference scores provide a summary of the extent of adaptation across blocks (as analyzed in more detail in the first set of analyses described in Predicting adaptation from cognitive/neuropsychological measures and Questionnaires section), more suitable for correlational analyses. We computed these difference scores for RTs on both valid and invalid trials. Specifically, we computed the difference in mean RTs between the MV and 50–50 blocks for each trial type. This yielded two delta scores (delta valid and delta invalid): a more positive delta indicates better adaptation, as it suggests that children are speeding up their RTs when transitioning from the MV to the 50–50 block (see the LMs in Table 1). Both deltas were centered before entering the statistical model.

## Results

Descriptive statistics for accuracy and RTs are reported in Table 2.

**Table 2:** Flanker and Addy task descriptive statistics

Group	Block	TrialType	Accuracy	RTs (s)
*A: Flanker task*				
ADHD	MC	Congruent	0.97 ± 0.18	1.02 ± 0.41
	MC	Incongruent	0.93 ± 0.26	1.15 ± 0.47
	50–50	Congruent	0.98 ± 0.13	0.96 ± 0.39
	50–50	Incongruent	0.93 ± 0.26	1.05 ± 0.43
TD	MC	Congruent	0.99 ± 0.10	0.95 ± 0.35
	MC	Incongruent	0.97 ± 0.17	1.07 ± 0.43
	50–50	Congruent	0.99 ± 0.09	0.89 ± 0.32
	50–50	Incongruent	0.98 ± 0.15	0.97 ± 0.39
*B: Addy task*				
ADHD	MV	Go valid	0.96 ± 0.19	0.96 ± 0.40
	MV	Go invalid	0.90 ± 0.30	1.02 ± 0.40
	MV	NoGo	0.92 ± 0.28	–
	50–50	Go valid	0.96 ± 0.20	1.03 ± 0.42
	50–50	Go invalid	0.95 ± 0.21	1.02 ± 0.41
	50–50	NoGo	0.93 ± 0.25	–
TD	MV	Go valid	0.99 ± 0.08	1.04 ± 0.37
	MV	Go invalid	0.93 ± 0.25	1.14 ± 0.39
	MV	NoGo	0.97 ± 0.17	–
	50–50	Go valid	0.91 ± 0.09	1.01 ± 0.38
	50–50	Go invalid	0.99 ± 0.12	1.02 ± 0.38
	50–50	NoGo	0.98 ± 0.14	–

Mean ± SD for accuracy and reaction times (RTs) on congruent/valid and incongruent/invalid trials for children with ADHD and TD under two block conditions (mostly congruent/valid, MC/MV, and 50–50) in the Flanker task (Panel A) and in the Addy task (Panel B). ADHD, attention deficit hyperactivity disorder; MC, most congruent; MV, mostly valid; TD, typically developing.

### Adaptation in the flanker task

#### Accuracy

**Figure 3:**
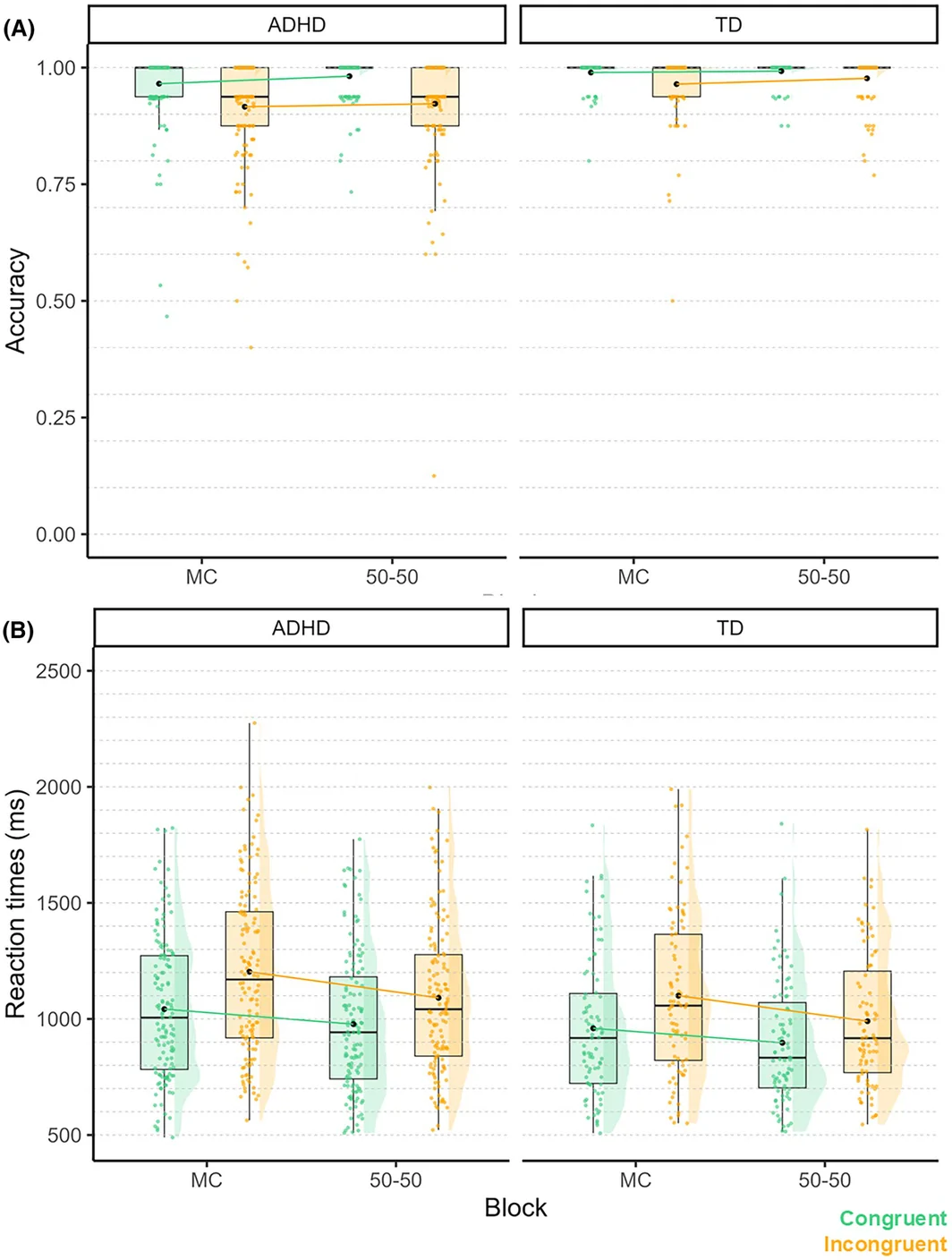
Accuracyand reaction times in the Flanker task. Panel A. Accuracy (*y*‐axis) is shown across blocks (mostly congruent, half congruent) on the *x*‐axis for congruent (green) and incongruent (orange) trials, separately for the ADHD (left) and TD (right) groups. Boxplots represent the median (line), interquartile range (box), and data range (whiskers); individual participant data points are overlaid. Panel B. Reaction times (*y*‐axis) are displayed using the same structure

See Table 3, Section A, for the full model results, and Figure 3A for a visual summary.

**Table 3:** Summary of Bayesian model results for accuracy (Section A) and reaction times (RTs; Section B) in the Flanker task

Effect	Estimate	95% HDI	PD
*Section A: Accuracy*			
TrialType (I)	**−0.98**	**−1.31, −0.66**	**>.99**
Block (50–50)	**0.39**	**0.04, 0.73**	.**99**
Group (TD)	**0.72**	**0.26, 1.14**	**>.99**
Age	**0.31**	**0.05, 0.59**	.**99**
Sex (M)	0.04	−0.35, 0.44	.57
TrialType (I) × Block (50–50)	−0.33	−0.71, 0.07	.95
TrialType (I) × Group (TD)	0.2	−0.25, 0.66	.81
Block (50–50) × Group (TD)	0.1	−0.39, 0.61	.65
TrialType (I) × Age	−0.08	−0.37, 0.20	.7
Block (50–50) × Age	0.02	−0.29, 0.33	.53
Group (TD) × Age	−0.23	−0.63, 0.20	.86
TrialType (I) × Block (50–50) × Group (TD)	0.17	−0.40, 0.70	.73
TrialType (I) × Block (50–50) × Age	0.03	−0.31, 0.39	.56
TrialType (I) × Group (TD) × Age	0.31	−0.16, 0.76	.91
Block (50–50) × Group (TD) × Age	−0.38	−0.85, 0.07	.95
TrialType (I) × Block (50–50) × Group (TD) × Age	0.18	−0.34, 0.70	.75
*Section B: Reaction times*			
TrialType (I)	**0.14**	**0.12, 0.16**	**>.99**
Block (50–50)	**−0.05**	**−0.07, −0.04**	**>.99**
Group (TD)	**−0.07**	**−0.12, −0.02**	**>.99**
Age	**−0.16**	**−0.19, −0.13**	**>.99**
Sex (M)	−0.03	−0.08, 0.02	. 85
preRT	**0.07**	**0.06, 0.08**	**>.99**
Acc(*n*−1) (1)	**−0.07**	**−0.10, −0.04**	**>.99**
Trial (*n*−1) (incongruent)	**−0.02**	**−0.03, −0.01**	**>.99**
TrialType (I) × Block (50–50)	**−0.03**	**−0.05, −0.01**	**>.99**
TrialType (I) × Group (TD)	0	−0.03, 0.03	.55
Block (50–50) × Group (TD)	0.01	−0.02, 0.03	.66
TrialType (I) × Age	0.01	−0.01, 0.03	.70
Block (50–50) × Age	−0.01	−0.03, 0.01	.88
Group (TD) × Age	0	−0.05, 0.05	.56
TrialType (I) × Block (50–50) × Group (TD)	−0.01	−0.04, 0.03	.66
TrialType (I) × Block (50–50) × Age	0.01	−0.01, 0.03	.76
TrialType (I) × Group (TD) × Age	−0.02	−0.05, 0.01	.86
Block (50–50) × Group (TD) × Age	0.02	0.001, 0.05	.96
Trialtype (I) × Block (50–50) × Group (TD) × Age	0	−0.04, 0.03	.58
*Post‐hoc contrasts*			
Block (MC): C − I	**−0.13**	**−0.15, −0.12**	**>.99**
Block (50–50): C − I	**−0.11**	**−0.12, −0.09**	**>.99**
TrialType (C): block (MC) − block (50–50)	**0.06**	**0.04, 0.07**	**>.99**
TrialType (I): block (MC) − block (50–50)	**0.08**	**0.07, 0.1**	**>.99**

Posterior mean estimates (log‐odds for accuracy and raw values for RTs) are presented along with their associated 95% highest density intervals (HDIs) and the probability of direction (PD). Interactions are indicated using the symbol **×.** Bolded results indicate strong evidence. MC, most congruent; TD, typically developing.

Overall, accuracy increased with age, suggesting developmental improvements in performance. Children from both groups were more accurate on congruent compared to incongruent trials, indicating a robust congruency effect.

Accuracy also improved from the MC to the 50:50 block, indicating a main effect of block, but there was no interaction between trial type and block, reflecting the absence of LWPC effect. There was a main effect of group, indicating that TD children outperformed their peers with ADHD, but group was not involved in any meaningful interaction, suggesting that learning‐based CC was not less effective in children with ADHD.

#### Reaction times

See Table 3, Section B, for the full model results, and Figure 3B for a visual summary.

Overall, there was a main effect of age, suggesting that RTs improved with increasing age. Additionally, we found the expected posterror slowing effect: slower responses following incongruent compared to congruent trials, and temporal correlations in RTs across consecutive trials.

Participants responded faster on congruent compared to incongruent trials, reflecting a clear congruency effect. RTs also decreased from the MC to the 50:50 block, indicating an overall speed improvement across blocks, and there was an interaction between block and trial type – also in line with the expected LWPC effect. This result suggests that the congruency effect was modulated by contextual difficulty, with a smaller effect observed in the 50:50 block compared to the MC block. Again, we also observed a main effect of group, with TD participants responding faster than their peers with ADHD, but no interaction involving group, indicating that learning‐based CC was not less effective in children with ADHD.

### Adaptation in the Addy task

#### Accuracy

**Figure 4:**
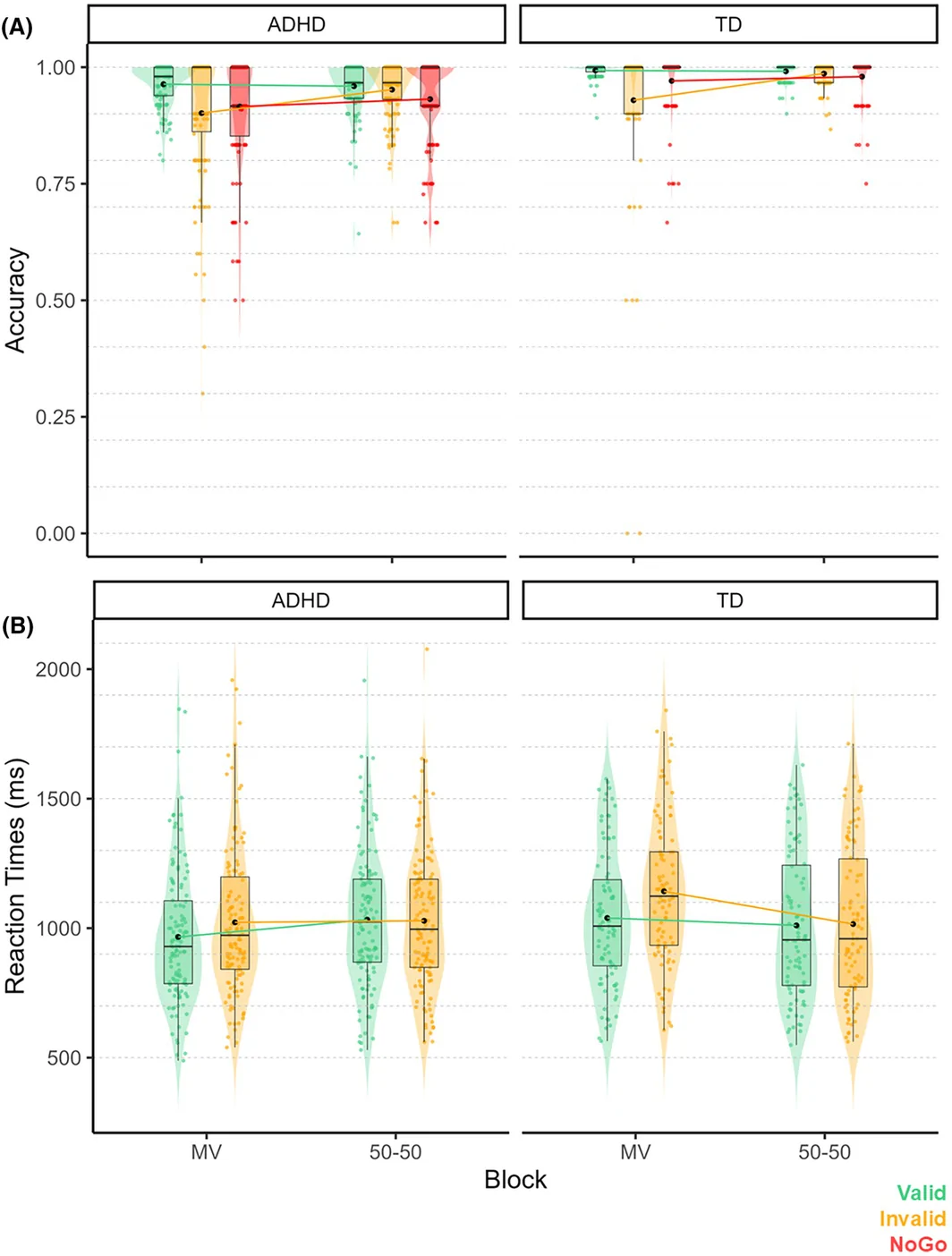
Accuracy and reaction time in the Addy task. Panel A. Accuracy (*y*‐axis) is shown across blocks (mostly valid, half valid) on the *x*‐axis for valid (green), invalid (orange), and noGo (red) trials, separately for the attention deficit hyperactivity disorder (left) and typically developing (right) groups. Boxplots represent the median (line), interquartile range (box), and data range (whiskers); individual participant data points are overlaid. Panel B. Reaction times (*y*‐axis) are displayed using the same structure

See Table 4, Section A, for the full model results, and Figure 4A for a visual summary.

**Table 4:** Summary of Bayesian model results for accuracy (Section A) and reaction times (RTs; Section B) in the Addy task

Effect	Estimate	95% HDI	PD
*Section A: Accuracy*			
TrialType (Go invalid)	**−0.58**	**−0.85, −0.29**	**>.99**
TrialType (NoGo)	**−0.68**	**−0.93, −0.45**	**>.99**
Block (50–50)	−0.09	−0.28, 0.12	.77
Group (TD)	**1.13**	**0.83, 1.43**	**>.99**
Age	**0.28**	**0.11, 0.45**	.**99**
Sex (M)	**−0.4**	**−0.67, −0.11**	.**99**
TrialType (Go invalid) × Block (50–50)	**0.5**	**0.19, 0.83**	.**99**
TrialType (NoGo) × Block (50–50)	0.2	−0.09, 0.49	.86
TrialType (Go invalid) × Group (TD)	−0.45	−0.84, −0.09	.97
TrialType (NoGo) × Group (TD)	−0.15	−0.5, 0.2	.75
block (50–50) × Group (TD)	0.14	−0.18, 0.48	.75
TrialType (Go invalid) × Age	−0.03	−0.27, 0.22	.57
TrialType (NoGo) × Age	0	−0.2, 0.21	.5
Block (50–50) × Age	−0.12	−0.3, 0.06	.86
Group (TD) × Age	0.05	−0.24, 0.33	.61
TrialType (Go invalid) × Block (50–50) × Group (TD)	0.28	−0.13, 0.72	.85
TrialType (NoGo) × Block (50–50) × Group (TD)	0.01	−0.44, 0.45	.51
TrialType (Go invalid) × Block (50–50) × Age	0.04	−0.24, 0.3	.58
TrialType (NoGo) × Block (50–50) × Age	−0.17	−0.42, 0.08	.86
TrialType (Go invalid) × Group (TD) × Age	0.06	−0.31, 0.42	.61
TrialType (NoGo) × Group (TD) × Age	−0.11	−0.45, 0.23	.69
block (50–50) × Group (TD) × Age	−0.1	−0.41, 0.23	.7
TrialType (Go invalid) × Block (50–50) × Group (TD) × Age	0.11	−0.29, 0.54	.67
TrialType (NoGo) × Block (50–50) × Group (TD) × Age	−0.04	−0.47, 0.4	.56
*Post hoc contrasts*			
Block (MV): Go valid–Go_invalid	**0.81**	**0.44, 1.2**	**>.99**
Block (MV): Go valid–NoGo	**0.76**	**0.45, 1.1**	**>.99**
Block (MV): Go invalid**–**NoGo	−0.05	−0.48, 0.41	.58
Block (50–50): Go valid**–**Go_invalid	0.17	−0.16, 0.48	.85
Block (50–50): Go valid–NoGo	**0.56**	**0.2, 0.93**	**>.99**
Block (50–50): Go invalid–NoGo	0.39	0, 0.77	.97
Go valid: block (MV)–block (50–50)	0.02	−0.26, 0.28	.56
Go invalid: block (MV)–block (50–50)	**−0.62**	**−1.05, −0.21**	**>.99**
NoGo: block (MV)–block (50–50)	−0.18	−0.55, 0.18	.84
*Section B: Reaction times*			
TrialType (Go invalid)	**0.07**	**0.05, 0.09**	**>.99**
BLOCK (50–50)	**0.07**	**0.04, 0.09**	**>.99**
Group (TD)	0.05	−0.01, 0.12	.95
Age	**−0.08**	**−0.12, −0.04**	**>.99**
Sex (m)	−0.03	−0.09, 0.03	.79
preRT	**0.13**	**0.12, 0.14**	**>.99**
Acc(*n*−1) (0)	**0.11**	**0.09, 0.12**	**>.99**
TrialType (*n*−1) (invalid)	**−0.01**	**−0.02, 0.001**	**>.99**
TrialType (*n*−1) (NoGo)	**−0.27**	**−0.29, −0.24**	**>.99**
TrialType (Go invalid) × Block (50–50)	**−0.07**	**−0.09, −0.05**	**>.99**
TrialType (Go invalid) × Group (TD)	0.03	0.001, 0.06	.97
Block (50–50) × Group (TD)	**−0.1**	**−0.13, −0.06**	**>.99**
TrialType (Go invalid) × Age	0	−0.02, 0.02	.59
Block (50–50) × Age	−0.01	−0.03, 0.01	.84
Group (td) × Age	0.05	0.001, 0.11	.97
TrialType (Go invalid) × Block (50–50) × Group (TD)	−0.03	−0.06, 0.01	.92
TrialType (Go invalid) × Block (50–50) × Age	0.01	−0.02, 0.03	.74
TrialType (Go invalid) × Group (TD) × Age	−0.03	−0.06, 0.001	.98
Block (50–50) × Group (TD) × Age	−0.02	−0.06, 0.01	.88
Trialtype (Go Invalid) × Block (50–50) × Group (TD) × Age1	0.01	−0.05, 0.01	.70
*Post hoc contrast*			
Block MV: Go valid–Go invalid	**−0.1**	**−0.11, −0.08**	**>.99**
Block 50–50: Go valid–Go invalid	**−0.03**	**−0.04, −0.02**	**>.99**
Go valid: block (MV)–block (50–50)	−0.01	−0.02, 0.01	0.75
Go invalid: block (MV)–block (50–50)	**0.06**	**0.04, 0.08**	**>.99**
ADHD: block (MV)–block (50–50)	**−0.03**	**−0.05, −0.01**	.**99**
TD: block (MV)–block (50–50)	**0.09**	**0.06, 0.11**	**>.99**
Block MV: ADHD–TD	**−0.08**	**−0.15, −0.01**	.**99**
Block 50–50: ADHD–TD	0.04	−0.04, 0.11	.83

Posterior mean estimates (log‐odds for accuracy and raw values for RTs) are presented along with their associated 95% highest density intervals (HDIs) and the probability of direction (PD). Interactions are indicated using the symbol **×.** Bolded results indicate strong evidence. ADHD, attention deficit hyperactivity disorder; MV, mostly valid; TD, typically developing.

Overall, there was the expected main effect of age, with older children being more accurate, and a main effect of sex, with females exhibiting higher overall accuracy than males. Children from both groups demonstrated higher accuracy on valid trials compared to invalid and NoGo trials. This effect interacted with block type: specifically, accuracy improved on invalid trials when children transitioned from the MV to the 50:50 block. Lastly, a main effect of group indicated that children with ADHD were generally less accurate than their TD peers, but group again did not interact with other variables, indicating that both groups improved to the same extent on invalid trials when moving from the MV to the 50:50 block.

#### Reaction times

See Table 4, Section B, for the full model results, and Figure 4B for a visual summary.

Overall, we found a main effect of age, with RTs decreasing as age increased. We also found the posterror slowing effect, slower responses following invalid and noGo trials compared to valid trials, and a temporal correlation in RTs across consecutive trials.

We observed a significant main effect of trial type, with children responding faster to invalid Go trials, as well as an interaction between trial type and block type, indicating that children from both groups became faster on invalid trials when transitioning from the MV to the 50:50 block, while RTs on valid trials remained stable. This led to a reduced validity effect (i.e., the difference between valid and invalid RTs) in the 50:50 block.

Critically, a Block × Group interaction revealed divergent patterns between groups: TD children showed faster responses in the 50:50 block compared to the MV block, whereas children with ADHD exhibited slower RTs. As a result, children with ADHD were faster than TD peers in the MV block, but no group differences were observed in the 50:50 block.

### Predicting adaptation in the Addy task

#### Addy task and questionnaires measures

See Table 5A for the full model results and Appendix S4, Figure S3 for a visual summary. We found no association between performance adaptation and the Questionnaires' composite index score. The only meaningful result was that the TD group – compared to the ADHD group – showed larger RTs modulation as a function of block (delta) in valid and invalid trials.

**Table 5:** Performance adaptation and questionnaires/neuropsychological scores

Response	Term	Estimate	95% HDI	PD
*A: Questionnaires' composite score*				
Delta valid	Q composite score	0.1	−0.18, 0.33	.71
**Delta valid**	**Group (TD)**	**0.6**	**0.11, 1.12**	**>.99**
Delta valid	age	0.1	−0.07, 0.28	.90
Delta invalid	Q composite score	0.2	−0.11, 0.41	.87
**Delta invalid**	**Group (TD)**	**0.6**	**0.05, 1.05**	.**98**
Delta invalid	age	0.04	−0.13, 0.22	.68
*B: NPS composite score*				
Delta valid	NPS composite score	0.33	−0.02, 0.71	.96
Delta valid	age	−0.04	−0.29, 0.18	.64
**Delta invalid**	**NPS composite score**	**0.43**	**0.08, 0.79**	.**99**
Delta invalid	age	−0.04	−0.27, 0.20	.64

The table presents the regression results for Delta Valid and Delta Invalid scores with the questionnaires' composite score (Panel A) and with the neuropsychological tests' composite score (Panel B). For each predictor, we report the estimated coefficient (β), the 95% highest density interval (HDI), and the probability of direction (PD). Bolded results indicate strong evidence.

#### Addy task and NPS tests in the ADHD group

**Figure 5:**
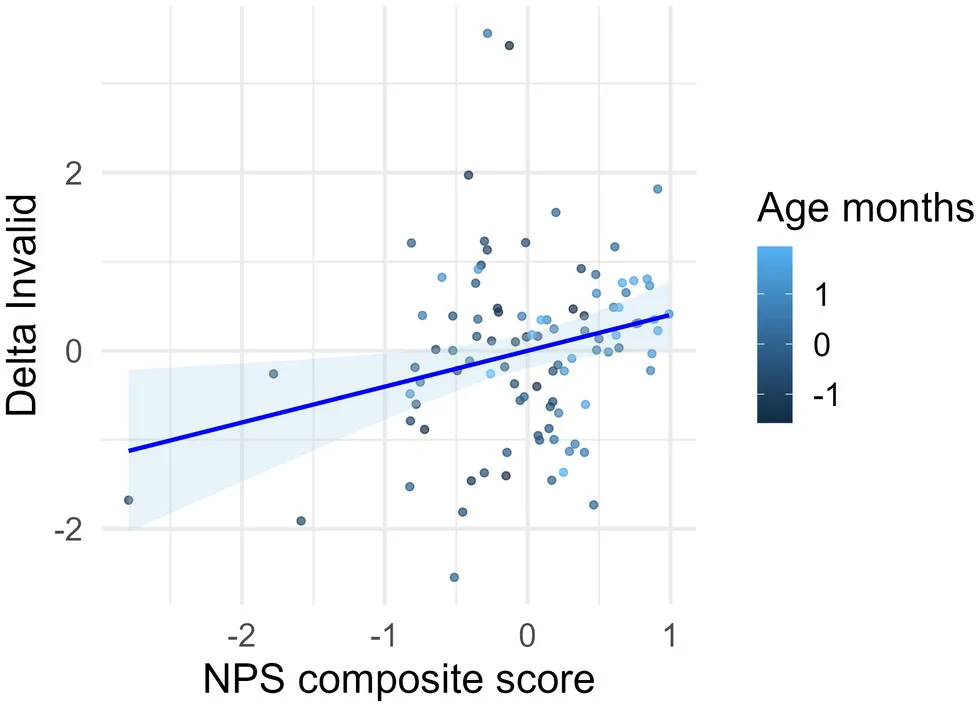
Delta invalid and neuropsychological (NPS) composite score. The plot displays scaled scores. On the *y*‐axis the Delta Invalid (i.e., mean reaction time of invalid trials in MC Block – 50:50 Block), and on the *x*‐axis the NPS composite score. Each point represents a participant, and the shadow around the regression line represents the standard error. The blue color scale is graded as a function of age in months, with lighter colors for older children and darker colors for younger children

See Table 5B for the full model results, and Figure 5 for a visual summary. We found a plausible positive association between the Neuropsychological index score and the delta invalid.

## Discussion

This multicentric study extends previous investigation of learning‐based CC by including children with ADHD (7–14 years). Two experimental tasks incorporating LWPC manipulations – a modified Flanker task (Gonthier et al., 2021) and a cued go‐noGo task (Addy task) – assessed how congruency and validity effects (i.e., expected better performance in congruent/valid compared to incongruent/invalid trials), respectively, are modulated based on contextual predictability.

### Learning‐based CC is not impaired in ADHD

The modified Flanker task and the Addy task allowed us to investigate how learning‐based CC evolves across typical and atypical development. Consistent with previous studies (Gonthier et al., 2021; Gonthier & Blaye, 2021), we found that TD children from preschool age can use contextual regularities to adaptively implement more reactive or more proactive control strategies. Remarkably, however, our results also showed that both TD children and children with ADHD, across all ages, displayed CC adaptation when facing increased contextual difficulty. Specifically, in the Flanker task both groups showed faster RTs on incongruent trials without accuracy loss from the mostly congruent (MC, less frequent incongruent trials) to the half congruent (50–50, more frequent incongruent trials) block. In the Addy task, both groups improved accuracy on invalid trials when moving from the MV to the half valid (50–50) block. In both cases, the effect counters the typical fatigue observed in similar tasks (which usually leads to lower performance in the second block) and provides robust evidence of CC adaptation corresponding to heightened control in the more difficult block. This LWPC effect can be interpreted as a form of shift toward proactive control (Gonthier et al., 2021), appearing to the same extent in both groups.

Notably, this is the first evidence that children with ADHD display LWPC effects similar to their TD peers. This aligns with prior findings showing that proactive CC based on implicit learning robustly appears in young TD children in an age range where explicit proactive control is not yet prevalent, suggesting that implicit implementation of CC is not as costly as explicit CC (Gonthier et al., 2021). Accordingly, we suggest that our results may reflect the relative preservation of automatic, context‐sensitive adjustments in children with ADHD, as opposed to their well‐documented difficulties in tasks that require explicit, strategic control. In other words, when control demands are implicit, children with ADHD can optimally modulate the implementation of CC depending on context, including proactive CC, contrasting with prior findings of impaired control modulation in this population (e.g., Cai et al., 2023; Cai, Chen, Szegletes, Supekar, & Menon, 2018; Sidlauskaite et al., 2020; Suarez et al., 2021).

### Learning‐based CC is hindered when multiple cognitive demands are at play

Most studies on learning‐based CC have used tasks targeting isolated cognitive mechanisms. Our second task, the cued go‐noGo (Addy task) requires multiple cognitive demands (i.e., allocation of visuospatial attention according to a preceding cue, while withholding responses to noGo trials). This created a task more reflective of real‐life demands (e.g., a child in a classroom must focus on the teacher's instructions while ignoring classroom distractions).

Remarkably, adaptation patterns differed between groups. Indeed, children in both groups improved accuracy on invalid trials when moving from the MV to the half valid (50–50) block, suggesting a shift toward proactive control. However, their speed‐accuracy trade‐offs diverged. TD children were faster in the second 50–50 block, showing specifically a speeding up on invalid trials. Conversely, children with ADHD were slower overall, maintaining RTs on invalid trials but slowing down on valid ones, suggesting less efficient control modulation. In other words, TD children showed a better speed‐accuracy balance than those with ADHD, aligning with past findings of altered trade‐offs in ADHD (Mohamed, Börger, Geuze, & van der Meere, 2016; van der Meere, 2005).

These results suggest that ADHD‐related difficulties are not due to impaired learning‐based CC per se, but rather to challenges in applying it under complex, multi‐demand conditions. This underscores the importance of using more ecologically valid experimental designs to understand how CC functions in real‐life contexts, especially in populations with neurodevelopmental disorders like ADHD.

### Learning‐based CC might relate to explicit CC

Some studies suggest that implicit control is dissociated from explicit control (Gonthier et al., 2021). Other studies indicate an association between the two (Mento et al., 2022). Anyway, a direct comparison between implicit and explicit CC mechanisms testing is still challenging. In this study, we explored whether task adaptation (RTs) was linked to scores from parental questionnaires and NPS tests, conventionally based on an explicit demand of CC. Interestingly, we found that better performance on a composite NPS score predicted stronger adaptation to invalid trials – a key group difference – while no link was found with questionnaire scores. This might suggest a connection between learning‐based (i.e., task adaptation) and explicit (i.e., NPS composite score) CC. However, as this was an exploratory finding, it should be interpreted cautiously and confirmed by future research.

### Limitations

Some limitations need to be acknowledged. First, we could not include a mostly incongruent block or counterbalance block order due to time and resource constraints. Including these in future studies could clarify how different forms of contextual predictability affect learning‐based CC in ADHD.

Second, the design of the cued go‐noGo task (Addy task) does not distinguish whether the effects are due to cue‐target learning difficulty (compared to the Flanker task, which only presents an LWPC manipulation) or combined demands on attentional control and response inhibition. Future research should isolate these contributions to better understand the mechanisms underlying learning‐based CC, particularly in ADHD.

Finally, it should be noted that children in the TD group may have had undiagnosed learning or attention difficulties, which are relatively common in school‐age populations (5%–6%; Morsanyi, van Bers, McCormack, & McGourty, 2018; Salari et al., 2023) and could partly account for the absence of group differences observed in the Flanker task.

## Ethical considerations

All parents provided written consent, and children provided oral assent after procedure explanation, following Italian ethical guidelines for research (Associazione Italiana di Psicologia, 2022). All experimental procedures were approved by the Ethics Committee (School of Psychology – Padova University, protocol no. 4920 of August 8, 2022) and followed the principles expressed in the Declaration of Helsinki.

**Key points**

**What is known?**

- Children with ADHD often show difficulties with cognitive control (CC), but little is known about their ability to adapt CC based on contextual learning.

**What is new?**

- This study shows that children with ADHD, like their TD peers, can use contextual regularities to adapt CC in low‐demand settings.
- In tasks that require multiple cognitive processes (e.g., attentional control and inhibition), children with ADHD show reduced adaptation efficiency. Neuropsychological performance emerged as a potential key mechanism explaining group differences in adaptive control.

**What is relevant?**

- Findings highlight the importance of using ecologically valid tasks and suggest that interventions for ADHD should target flexible CC in real‐life, multidemand contexts.

## Supporting information


**Appendix S1.** Study overview and sample characteristics.
**Figure S1.** Visual representation of the clinical centers and schools involved in the ‘CALM‐1’ multicentric study.
**Figure S2.** Raincloud plot and boxplot of participants' age (years) distribution in the final TD group (orange) and ADHD group (pink) for the Flanker (left) and Addy (right) tasks.
**Appendix S2.** Sample selection and measures.
**Table S1.** Summary of participant exclusions by task (Flanker and Addy).
**Table S2.** Description and descriptive statistics of the neuropsychological (NPS) tests.
**Table S3.** Description and descriptive statistics for parent‐report questionnaires.
**Appendix S3.** Full statistical models' details.
**Table S4.** Prior distribution summary used in the model_commissions_Flanker.
**Table S5.** Prior distribution summary used in the model_RT_Flanker.
**Table S6.** Prior distribution summary used in the model_commissions_Addy.
**Table S7.** Prior distribution summary used in the model_RT_Addy.
**Table S8.** Prior distribution summary used in the model_quest_Addy.
**Table S9.** Prior distribution summary used in the model_NPS_Addy.
**Appendix S4.** Frequentist statistical analyses results.
**Table S10.** Summary of frequentist model results for accuracy (Section A) and reaction times (RTs; Section B).
**Table S11.** Summary of frequentist model results for accuracy (Section A) and reaction times (RTs; Section B).
**Table S12.** Summary of frequentist model results for Addy's delta scores and questionnaires composite score.
**Table S13.** Summary of frequentist model results for Addy's delta scores and NPS composite score in ADHD.
**Figure S3.** Delta valid and delta invalid and group.

## Data Availability

The data that support the findings of this study are openly available in OSF at https://osf.io/gae2c/?view_only=97462614f1b74214a8b451119428c604.

## Endnote

1 Note that the items sets for inducer and diagnostic trials were not counterbalanced (up/down arrows were always used for inducer trials and left/right for diagnostic trials). Given that responding to up/down arrows is slower and given that PC effects do not show a major interaction with the arrow direction (Gonthier et al., 2021), not counterbalancing the item sets made the design more robust, especially in the context of a group comparison (i.e., the measure of interest for all participants was always left/right arrows in a MC vs. 50–50 block), without substantial loss of generalizability. This design choice is reflected in slightly slower RTs overall for diagnostic compared to inducer trials.

## References

[jcpp70162-bib-0001] Abrahamse, E. , Braem, S. , Notebaert, W. , & Verguts, T. (2016). Grounding cognitive control in associative learning. Psychological Bulletin, 142, 693–728. 27148628 10.1037/bul0000047

[jcpp70162-bib-0002] Achenbach, T.M. , & Edelbrock, C. (1991). Child behavior checklist. Burlington (vt), 7(622), 371–392.

[jcpp70162-bib-0003] American Psychiatric Association, D , & American Psychiatric Association, D. S . (2013). Diagnostic and statistical manual of mental disorders: DSM‐5 (Vol. 5). Washington, DC: Author. Available from: https://www.academia.edu/download/38718268/csl6820_21.pdf

[jcpp70162-bib-0004] Associazione Italiana di Psicologia . (2022). Codice Etico per la Ricerca in Psicologia.

[jcpp70162-bib-0005] Baayen, R.H. , & Milin, P. (2010). Analyzing reaction times. International Journal of Psychological Research, 3, 12–28.

[jcpp70162-bib-0006] Baddeley, A.D. , & Hitch, G. (1974). Working memory. In G.H. Bower (Ed.), Psychology of learning and motivation (Vol. 8, pp. 47–89). New York, NY, USA: Academic Press.

[jcpp70162-bib-0007] Barnes, K.A. , Howard, J.H. , Howard, D.V. , Kenealy, L. , & Vaidya, C.J. (2010). Two forms of implicit learning in childhood ADHD. Developmental Neuropsychology, 35, 494–505. 20721771 10.1080/87565641.2010.494750 PMC2925298

[jcpp70162-bib-0008] Bottesi, G. , Noventa, S. , Freeston, M.H. , & Ghisi, M. (2019). Seeking certainty about intolerance of uncertainty: Addressing old and new issues through the intolerance of uncertainty scale‐revised. PLoS One, 14, e0211929. 30742694 10.1371/journal.pone.0211929 PMC6370219

[jcpp70162-bib-0009] Braem, S. , Bugg, J.M. , Schmidt, J.R. , Crump, M.J. , Weissman, D.H. , Notebaert, W. , & Egner, T. (2019). Measuring adaptive control in conflict tasks. Trends in Cognitive Sciences, 23, 769–783. 31331794 10.1016/j.tics.2019.07.002 PMC6699878

[jcpp70162-bib-0010] Braem, S. , & Egner, T. (2018). Getting a grip on cognitive flexibility. Current Directions in Psychological Science, 27, 470–476. 30555214 10.1177/0963721418787475 PMC6291219

[jcpp70162-bib-0011] Braver, T.S. (2012). The variable nature of cognitive control: A dual mechanisms framework. Trends in Cognitive Sciences, 16, 106–113. 22245618 10.1016/j.tics.2011.12.010 PMC3289517

[jcpp70162-bib-0012] Bugg, J.M. , & Crump, M.J. (2012). In support of a distinction between voluntary and stimulus‐driven control: A review of the literature on proportion congruent effects. Frontiers in Psychology, 3, 367. 23060836 10.3389/fpsyg.2012.00367 PMC3459019

[jcpp70162-bib-0013] Bürkner, P.‐C. (2017). brms: An R package for Bayesian multilevel models using Stan. Journal of Statistical Software, 80, 1–28.

[jcpp70162-bib-0014] Cai, W. , Chen, T. , Szegletes, L. , Supekar, K. , & Menon, V. (2018). Aberrant time‐varying cross‐network interactions in children with attention‐deficit/hyperactivity disorder and the relation to attention deficits. Biological Psychiatry: Cognitive Neuroscience and Neuroimaging, 3, 263–273. 29486868 10.1016/j.bpsc.2017.10.005 PMC5833018

[jcpp70162-bib-0015] Cai, W. , Warren, S.L. , Duberg, K. , Yu, A. , Hinshaw, S.P. , & Menon, V. (2023). Both reactive and proactive control are deficient in children with ADHD and predictive of clinical symptoms. Translational Psychiatry, 13, 1–12. 37236924 10.1038/s41398-023-02471-w PMC10220086

[jcpp70162-bib-0016] Castellanos, F.X. , Sonuga‐Barke, E.J. , Milham, M.P. , & Tannock, R. (2006). Characterizing cognition in ADHD: Beyond executive dysfunction. Trends in Cognitive Sciences, 10, 117–123. 16460990 10.1016/j.tics.2006.01.011

[jcpp70162-bib-0017] Chiu, Y.‐C. , & Egner, T. (2019). Cortical and subcortical contributions to context‐control learning. Neuroscience & Biobehavioral Reviews, 99, 33–41. 30685484 10.1016/j.neubiorev.2019.01.019 PMC6399056

[jcpp70162-bib-0018] Comer, J.S. , Roy, A.K. , Furr, J.M. , Gotimer, K. , Beidas, R.S. , Dugas, M.J. , & Kendall, P.C. (2009). The intolerance of uncertainty scale for children: A psychometric evaluation. Psychological Assessment, 21, 402–411. 19719351 10.1037/a0016719 PMC2952545

[jcpp70162-bib-0019] Conners, C.K. , Sitarenios, G. , Parker, J.D. , & Epstein, J.N. (1998). The revised Conners' Parent Rating Scale (CPRS‐R): Factor structure, reliability, and criterion validity. Journal of Abnormal Child Psychology, 26, 257–268. 9700518 10.1023/a:1022602400621

[jcpp70162-bib-0020] Davidovitch, M. , Koren, G. , Fund, N. , Shrem, M. , & Porath, A. (2017). Challenges in defining the rates of ADHD diagnosis and treatment: Trends over the last decade. BMC Pediatrics, 17, 218. 29284437 10.1186/s12887-017-0971-0 PMC5747128

[jcpp70162-bib-0021] Diamond, A. (2013). Executive functions. Annual Review of Psychology, 64, 135–168. 10.1146/annurev-psych-113011-143750 PMC4084861 23020641

[jcpp70162-bib-0022] Diamond, A. (2020). Executive functions. In A. Gallagher , C. Bulteau , D. Cohen , & J.L. Michaud (Eds.), Handbook of clinical neurology (Vol. 173, pp. 225–240). Amsterdam, The Netherlands: Elsevier. 10.1016/B978-0-444-64150-2.00020-4 32958176

[jcpp70162-bib-0023] Dunn, T.J. , Baguley, T. , & Brunsden, V. (2014). From alpha to omega: A practical solution to the pervasive problem of internal consistency estimation. British Journal of Psychology, 105, 399–412. 24844115 10.1111/bjop.12046

[jcpp70162-bib-0024] Egner, T. (2014). Creatures of habit (and control): A multi‐level learning perspective on the modulation of congruency effects. Frontiers in Psychology, 5, 1247. 25414679 10.3389/fpsyg.2014.01247 PMC4222221

[jcpp70162-bib-0025] Gioia, G.A. , Isquith, P.K. , Guy, S.C. , & Kenworthy, L. (2015). BRIEF‐2: Behavior rating inventory of executive function, second edition. Lutz, FL, USA: Psychological Assessment Resources.

[jcpp70162-bib-0026] Gonthier, C. , Ambrosi, S. , & Blaye, A. (2021). Learning‐based before intentional cognitive control: Developmental evidence for a dissociation between implicit and explicit control. Journal of Experimental Psychology: Learning, Memory, and Cognition, 47, 1660–1685. 33764124 10.1037/xlm0001005

[jcpp70162-bib-0027] Gonthier, C. , & Blaye, A. (2021). Preschoolers are capable of fine‐grained implicit cognitive control: Evidence from development of the context‐specific proportion congruency effect. Journal of Experimental Child Psychology, 210, 105211. 34157498 10.1016/j.jecp.2021.105211

[jcpp70162-bib-0028] Grant, D.A. , & Berg, E. (1948). A behavioral analysis of degree of reinforcement and ease of shifting to new responses in a Weigl‐type card‐sorting problem. Journal of Experimental Psychology, 38(4), 404. 18874598 10.1037/h0059831

[jcpp70162-bib-0029] Gratton, G. , Coles, M.G. , & Donchin, E. (1992). Optimizing the use of information: Strategic control of activation of responses. Journal of Experimental Psychology: General, 121, 480–506. 1431740 10.1037//0096-3445.121.4.480

[jcpp70162-bib-0030] Heaton, R.K. (1981). Wisconsin card sorting test manual. Lutz, FL: Psychological Assessment Resources.

[jcpp70162-bib-0060] Horga, G. , Maia, T.V. , Wang, P. , Wang, Z. , Marsh, R. , & Peterson, B.S. (2011). Adaptation to conflict via context‐driven anticipatory signals in the dorsomedial prefrontal cortex. Journal of Neuroscience, 31(45), 16208–16216. 22072672 10.1523/JNEUROSCI.2783-11.2011 PMC3244974

[jcpp70162-bib-0031] Huang‐Pollock, C.L. , Maddox, W.T. , & Tam, H. (2014). Rule‐based and information‐integration perceptual category learning in children with attention‐deficit/hyperactivity disorder. Neuropsychology, 28, 594–604. 24635709 10.1037/neu0000075 PMC4104575

[jcpp70162-bib-0032] Korkman, M. , Kirk, U. , & Kemp, S. (2007). NEPSY‐II‐Second edition. Available from: https://www.medeacom.org/slides/2017/05%20‐%20Maggio/0522%20‐%20CSR/09_Nepsy_II_Serra_2017.pdf

[jcpp70162-bib-0033] Laasonen, M. , Väre, J. , Oksanen‐Hennah, H. , Leppämäki, S. , Tani, P. , Harno, H. , … & Cleeremans, A. (2014). Project DyAdd: Implicit learning in adult dyslexia and ADHD. Annals of Dyslexia, 64, 1–33. 24162872 10.1007/s11881-013-0083-y

[jcpp70162-bib-0034] Lakens, D. (2022). Sample size justification. Collabra: Psychology, 8, 33267.

[jcpp70162-bib-0035] Lenth, R. (2020). emmeans: Estimated marginal means, aka least‐squares means (Version 1.5. 2‐1) [R package].

[jcpp70162-bib-0036] Luman, M. , Tripp, G. , & Scheres, A. (2010). Identifying the neurobiology of altered reinforcement sensitivity in ADHD: A review and research agenda. Neuroscience & Biobehavioral Reviews, 34, 744–754. 19944715 10.1016/j.neubiorev.2009.11.021

[jcpp70162-bib-0037] Makowski, D. , Ben‐Shachar, M. , & Lüdecke, D. (2019). bayestestR: Describing effects and their uncertainty, existence and significance within the Bayesian framework. The Journal of Open Source Software, 4, 1541.

[jcpp70162-bib-0038] Marzocchi, G.M. , Re, A.M. , & Cornoldi, C. (2010). BIA. Batteria italiana per l'ADHID per la valutazione dei bambini con deficit di attenzione‐iperattività. Con DVD e CD‐ROM. Edizioni Erickson. Available from: https://books.google.com/books?hl=it&lr=&id=8foRIr_1VdAC&oi=fnd&pg=PA6&dq=marzocchi+et+al+2010+Batteria+italiana+adhd&ots=lmXM3qIams&sig=XrdS2xCqMUl2rt94md8DE6NTmr4.

[jcpp70162-bib-0039] Mento, G. , Toffoli, L. , Della Longa, L. , Farroni, T. , Del Popolo Cristaldi, F. , & Duma, G.M. (2022). Adaptive cognitive control in prematurely born children: An HD‐EEG investigation. Brain Sciences, 12, 1074. 36009137 10.3390/brainsci12081074 PMC9406101

[jcpp70162-bib-0040] Miyake, A. , & Friedman, N.P. (2012). The nature and organization of individual differences in executive functions: Four general conclusions. Current Directions in Psychological Science, 21, 8–14. 22773897 10.1177/0963721411429458 PMC3388901

[jcpp70162-bib-0041] Mohamed, S.M.H. , Börger, N.A. , Geuze, R.H. , & van der Meere, J.J. (2016). Post‐error adjustments and ADHD symptoms in adults: The effect of laterality and state regulation. Brain and Cognition, 108, 11–19. 27429094 10.1016/j.bandc.2016.06.006

[jcpp70162-bib-0042] Morsanyi, K. , van Bers, B.M.C.W. , McCormack, T. , & McGourty, J. (2018). The prevalence of specific learning disorder in mathematics and comorbidity with other developmental disorders in primary school‐age children. British Journal of Psychology, 109, 917–940. 29974939 10.1111/bjop.12322

[jcpp70162-bib-0059] Mueller, S.T. , & Piper, B.J. (2014). The psychology experiment building language (PEBL) and PEBL test battery. Journal of Neuroscience Methods, 222, 250–259. 24269254 10.1016/j.jneumeth.2013.10.024 PMC3897935

[jcpp70162-bib-0043] Nigg, J.T. (2017). Getting ahead of ADHD: What next‐generation science says about treatments that work—and how you can make them work for your child. New York, NY, USA: Guilford Publications.

[jcpp70162-bib-0044] Nobile, M. , Alberti, B. , & Zuddas, A. (2007). CRS‐R. Conners' rating scales—Revised. Manuale. Firenze, Italia: Giunti Editore.

[jcpp70162-bib-0045] Norman, D.A. , & Shallice, T. (1986). Attention to action. In Consciousness and self‐regulation (pp. 1–18). Boston, MA: Springer.

[jcpp70162-bib-0046] Polanczyk, G. , De Lima, M.S. , Horta, B.L. , Biederman, J. , & Rohde, L.A. (2007). The worldwide prevalence of ADHD: A systematic review and Metaregression analysis. American Journal of Psychiatry, 164, 942–948. 17541055 10.1176/ajp.2007.164.6.942

[jcpp70162-bib-0047] R Core Team . (2020). R: A language and environment for statistical computing. Vienna: R Foundation for Statistical Computing. https://cir.nii.ac.jp/crid/1370298755636824325.

[jcpp70162-bib-0048] Rabbitt, P.A. (1966). Errors and error correction in choice‐response tasks. Journal of Experimental Psychology, 71, 264–272. 5948188 10.1037/h0022853

[jcpp70162-bib-0049] Raven, J.C. (1990). Court, JH, & Raven, J. (1995). Coloured progressive matrices, 10–11.

[jcpp70162-bib-0050] Salari, N. , Ghasemi, H. , Abdoli, N. , Rahmani, A. , Shiri, M.H. , Hashemian, A.H. , … & Mohammadi, M. (2023). The global prevalence of ADHD in children and adolescents: A systematic review and meta‐analysis. Italian Journal of Pediatrics, 49, 48. 37081447 10.1186/s13052-023-01456-1 PMC10120242

[jcpp70162-bib-0051] Schneider, W. , Eschman, A. , & Zuccolotto, A. (2002). E‐Prime computer software and manual. Pittsburgh, PA, USA: Psychology Software Tools.

[jcpp70162-bib-0052] Sidlauskaite, J. , Dhar, M. , Sonuga‐Barke, E. , & Wiersema, J.R. (2020). Altered proactive control in adults with ADHD: Evidence from event‐related potentials during cued task switching. Neuropsychologia, 138, 107330. 31887312 10.1016/j.neuropsychologia.2019.107330

[jcpp70162-bib-0053] Sonuga‐Barke, E.J. , & Sergeant, J.A. (2005). The neuroscience of ADHD: Multidisciplinary perspectives on a complex developmental disorder. Developmental Science, 8, 103–104. 15720367 10.1111/j.1467-7687.2005.00396.x

[jcpp70162-bib-0054] Suarez, I. , De los Reyes Aragón, C. , Grandjean, A. , Barceló, E. , Mebarak, M. , Lewis, S. , … & Casini, L. (2021). Two sides of the same coin: ADHD affects reactive but not proactive inhibition in children. Cognitive Neuropsychology, 38, 349–363. 35209797 10.1080/02643294.2022.2031944

[jcpp70162-bib-0055] van der Meere, J.J. (2005). ADHD and state regulation. In D. Gozal & D.L. Molfese (Eds.), Attention deficit hyperactivity disorder: From genes to animal models to patients (pp. 413–433). Totowa, NJ, USA: Humana Press.

[jcpp70162-bib-0056] van Hulst, B.M. , de Zeeuw, P. , Vlaskamp, C. , Rijks, Y. , Zandbelt, B.B. , & Durston, S. (2018). Children with ADHD symptoms show deficits in reactive but not proactive inhibition, irrespective of their formal diagnosis. Psychological Medicine, 48, 2515–2521. 29415788 10.1017/S0033291718000107 PMC6190063

[jcpp70162-bib-0057] Willcutt, E.G. , Doyle, A.E. , Nigg, J.T. , Faraone, S.V. , & Pennington, B.F. (2005). Validity of the executive function theory of attention‐deficit/hyperactivity disorder: A meta‐analytic review. Biological Psychiatry, 57, 1336–1346. 15950006 10.1016/j.biopsych.2005.02.006

[jcpp70162-bib-0058] Xu, G. , Strathearn, L. , Liu, B. , Yang, B. , & Bao, W. (2018). Twenty‐year trends in diagnosed attention‐deficit/hyperactivity disorder among US children and adolescents, 1997‐2016. JAMA Network Open, 1, e181471. 30646132 10.1001/jamanetworkopen.2018.1471 PMC6324288

