# Supplementary material for: Learning‐based cognitive control in ADHD: a multicentric study
Source: J Child Psychol Psychiatry. 2026 May 5;67(10):1624–38. doi: 10.1111/jcpp.70162 (PMC13535711; doi:10.1111/jcpp.70162)
Supplement: Supplementary file 1 [file JCPP-67-1624-s001.docx]

**Learning-based cognitive control in ADHD:**

**a multicentric study**

**Supporting Information**

**Appendix S1. Study Overview and Sample Characteristics**

**Figure S1.** Visual representation of the clinical centers and schools involved in the “CALM-1” multicentric study. Veneto region (purple pinpoint): IRCCS E.Medea ‘La Nostra Famiglia’ in Conegliano, Treviso, Vicenza, Padova and Oderzo; Studio di Psicologia e Psicoterapia per l’età evolutiva (Venezia); Studio La Fenice (Venezia); Studio Incore (Padova), centro medico Rindola (Vicenza). Emilia-Romagna region (pink pinpoint): Studio Il Piccolo Principe (Ferrara). Liguria region (light pink pinpoint): Studio Logopedico Psicologico Il Timone (La Spezia). Marche region (blue pinpoint): Maria Montessori Nursery School (Ancona); Raffaello Sanzio Primary School (Ancona). Sicily region (light blue pinpoint): Antonio Gramsci Middle School (Palermo); Giovanni Falcone Comprehensive Institute (Palermo).

**Figure S2.** Raincloud plot and boxplot of participants’ age (years) distribution in the final TD group (orange) and ADHD group (pink) for the Flanker (left) and Addy (right) tasks. To note, participants of both tasks are drawn from the same initial sample and data cleaning procedures led to different sample sizes.

**Appendix S2. Sample Selection and Measures**

| **Exclusion Reason** | **Flanker (n)** | **Addy (n)** |
| --- | --- | --- |
| Technical issues | 18 | 2 |
| Sensory/neurological conditions (epilepsy, fetal alcohol syndrome) | 2 | 2 |
| Low non-verbal reasoning (CPM < 5th percentile or WISC-IV < 70) | 16 | 15 |
| Incomplete task (<90% trials completed) | 10 | 0 |
| Low overall accuracy (<60%) | 5 | 41 |
| TD children under 7 years (to match ADHD age range) | 37 | 30 |
| **Total Excluded** | **88** | **90** |

**Table S1. Summary of participant exclusions by task (Flanker and Addy).** Reasons for exclusion included: technical issues (e.g., corrupted data file); sensory or neurological conditions; low non-verbal reasoning (<5th percentile of their age group) abilities as assessed using the Coloured Progressive Matrices [(CPM, Belacchi et al., 2008; Raven, 1990)](https://www.zotero.org/google-docs/?Pab2eO) or - when available - low score (<70) in at least one of the Wechsler Intelligence Scale for Children (WISC-IV, [Wechsler, 2012)](https://www.zotero.org/google-docs/?u7M2r5) composite scores (i.e., Verbal Comprehension Index, Visual Spatial Index, Working Memory Index, Processing Speed Index); incomplete task performance; low accuracy; and age mismatch.

# Anamnestic Questionnaire

## Versione Italiana

1. Sono presenti diagnosi relative a problematiche dello sviluppo (es., autismo, iperattività, ritardo di linguaggio, problemi di apprendimento, disabilità) diagnosticate da un/a professionista?
 ☐ Sì ☐ No
 Se sì, specificare quale/i diagnosi relativa/e allo sviluppo è/sono presente/i:
 ________________________________________

2. Sono presenti altri problemi di natura medica diagnosticati da un/a professionista (ad esempio, epilessia)?
 ☐ Sì ☐ No
 Se sì, specificare quale/i problema/i di natura medica è/sono presente/i:
 ________________________________________

3. Il/La bambino/a assume farmaci quotidianamente (es. metilfenidato)?
 ☐ Sì ☐ No
 Se sì, specificare quale/i farmaco/i assume e in che dosaggio (es. metilfenidato, 0.2 mg al giorno):
 ________________________________________

4. Ci sono parenti di primo o secondo grado (es. genitori, zii, cugini) che presentano o hanno presentato problematiche dello sviluppo (es. autismo, iperattività, ritardo di linguaggio, problemi di apprendimento, disabilità)?
 ☐ Sì ☐ No
 Se sì, specificare quali problematiche dello sviluppo:
 ________________________________________

5. Il/La bambino/a è nato/a:
 ☐ Pretermine (< 37 settimane)
 ☐ A termine (37–41 settimane)
 ☐ Post-termine (> 42 settimane)
 Numero di settimane di gravidanza al momento del parto: ________ settimane
 Peso alla nascita (in grammi): ________ g

6. Il/La bambino/a presenta problemi connessi al sonno (ad esempio, difficoltà ad addormentarsi, risvegli notturni, sonno disturbato)?
 ☐ Sì ☐ No

7. Il rendimento scolastico è:
 ☐ Brillante ☐ Nella norma ☐ Un po’ scarso ☐ Molto scarso
 In quali ambiti ha maggiore difficoltà?
 ☐ Lettura ☐ Calcolo ☐ Studio ☐ Nessuno

## English Version

1. Has the child received any diagnoses related to developmental conditions (e.g., autism, hyperactivity, language delay, learning disorders, intellectual disability) from a qualified professional?
 ☐ Yes ☐ No
 If yes, please specify the developmental diagnosis(es):
 ________________________________________

2. Has the child been diagnosed with any other medical conditions by a qualified professional (e.g., epilepsy)?
 ☐ Yes ☐ No
 If yes, please specify the medical condition(s):
 ________________________________________

3. Does the child take any daily medication (e.g., methylphenidate)?
 ☐ Yes ☐ No
 If yes, please specify the medication(s) and dosage (e.g., methylphenidate, 0.2 mg per day):
 ________________________________________

4. Are there first- or second-degree relatives (e.g., parents, uncles/aunts, cousins) who currently have or previously had developmental conditions (e.g., autism, hyperactivity, language delay, learning disorders, intellectual disability)?
 ☐ Yes ☐ No
 If yes, please specify the developmental condition(s):
 ________________________________________

5. The child was born:
 ☐ Preterm (< 37 weeks)
 ☐ Full term (37–41 weeks)
 ☐ Post-term (> 42 weeks)
 Gestational age at birth (in weeks): ________ weeks
 Birth weight (in grams): ________ g

6. Does the child have sleep-related difficulties (e.g., difficulty falling asleep, night awakenings, restless sleep)?
 ☐ Yes ☐ No

7. The child’s school performance is:
 ☐ Excellent ☐ Average ☐ Somewhat below average ☐ Poor
 In which areas does the child experience the greatest difficulty?
 ☐ Reading ☐ Mathematics ☐ Studying ☐ None

**Anamnestic questionnaire.** Items 1 and 2 of the survey were used as exclusion criteria, ensuring that participants in the TD group did not present a history of diagnosed neurodevelopmental or neurological/psychiatric conditions. The remaining items were included to collect broader anamnestic and contextual information as part of the lab’s standard assessment protocol. However, these variables were not used for inclusion/exclusion decisions in the present study, nor were they included in the current analyses, as they fall outside the scope of this paper. Accordingly, group classification was based on a categorical criterion, whereby only participants with a clear clinical diagnosis were included in the clinical group, and all others meeting the exclusion criteria were assigned to the TD group.

| **Test** | **Cognitive Function** | **Description** | **Considered scales** | **Mean ± SD** |
| --- | --- | --- | --- | --- |
| **Coloured Progressive Matrices (CPM)** | Non-verbal reasoning | Assesses abstract reasoning through pattern completion tasks. | Total score | 28 ± 4.47 |
| **Numerical Stroop (BIA-R)** | Interference control | Participants report the number of digits shown, ignoring their actual value. | Numerical Stroop errors | 3.27 ± 3.04 |
|  |  |  | Numerical Stroop time (seconds) | 133.88 ± 41.1 |
| **NEPSY-II Inhibition subtest** | Verbal inhibition | Requires naming shapes based on conflicting rules. | Nepsy Inhibition errors | 4.87 ± 3.99 |
|  |  |  | Nepsy Inhibition time (seconds) | 87.6 ± 34.91 |
| **Wisconsin Card Sorting Test (WCST, 64-card)** | Cognitive flexibility | Measures rule discovery and shifting in response to feedback. | WCST Perseverative errors | 11.45 ± 6.7 |
|  |  |  | WCST Perseverative responses | 22.08 ± 7.65 |

**Table S2. Description and descriptive statistics of the Neuropsychological (NPS) tests.** The table reports the description of cognitive and NPS tasks used to assess non-verbal reasoning, interference control, verbal inhibition, and cognitive flexibility in the ADHD group. For each measure, mean ± standard deviation (SD) are reported.

| **Questionnaire** | **Description** |  | **Scale** | **ADHD** | **TD** |
| --- | --- | --- | --- | --- | --- |
| **Conners’ Parent Rating Scale – Revised (CPRS-R)** | Attention and behavior problems: assesses ADHD symptoms and general behavioral issues based on parental ratings. | | **CPRS Oppositivity** | 10.02 ± 5.97 | 4.9 ± 4.4 |
|  |  |  | **CPRS Cognitive Problems and Disattention** | 18.61 ± 8.18 | 4.79 ± 4.72 |
|  |  |  | **CPRS Hyperactivity** | 9.52 ± 6.64 | 2.17 ± 2.85 |
|  |  |  | **CPRS Anxiety/Shyness** | 5.6 ± 3.84 | 5.45 ± 4.86 |
|  |  |  | **CPRS Perfectionism** | 3.96 ± 2.96 | 3.14 ± 2.82 |
|  |  |  | **CPRS Social Problems** | 2.99 ± 2.9 | 0.97 ± 1.76 |
|  |  |  | **CPRS Psychosomatic Problems** | 2.27 ± 3.45 | 0.93 ± 1.77 |
|  |  |  | **CPRS ADHD index** | 19.56 ± 8.58 | 4.9 ± 4.48 |
|  |  |  | **CPRS Impulsivity/Restlessness** | 9.61 ± 5.17 | 2.69 ± 2.9 |
|  |  |  | **CPRS Emotional Liability** | 2.08 ± 2.13 | 0.83 ± 1.2 |
|  |  |  | **CPRS Global Index** | 11.72 ± 6.44 | 3.52 ± 3.9 |
|  |  |  | **CPRS DSM-IV Disattention** | 14.81 ± 6.57 | 4.1 ± 3.75 |
|  |  |  | **CPRS DSM-IV Hyperactivity-impulsivity** | 11.68 ± 6.3 | 3.38 ± 3.6 |
|  |  |  | **CPRS DSM-IV Total** | 25.92 ± 11.88 | 7.48 ± 6.43 |
| **Child Behavior Checklist (CBCL)** | Emotional and behavioral functioning: evaluates internalizing and externalizing problems in everyday settings. | | **CBCL Anxiety/Depression** | 5.67 ± 3.59 | 3.9 ± 2.78 |
|  |  |  | **CBCL Withdrawal/Depression** | 2.62 ± 2.38 | 1.86 ± 1.87 |
|  |  |  | **CBCL Somatic Complaints** | 2.51 ± 2.83 | 1.45 ± 1.27 |
|  |  |  | **CBCL Social Problems** | 5.2 ± 3.4 | 2.24 ± 1.86 |
|  |  |  | **CBCL Thought Problems** | 3.66 ± 2.88 | 1.55 ± 1.5 |
|  |  |  | **CBCL Attention Problems** | 9.57 ± 3.81 | 2.55 ± 2.35 |
|  |  |  | **CBCL Rule-breaking Behavior** | 3.26 ± 2.75 | 1.24 ± 0.95 |
|  |  |  | **CBCL Aggressive Behavior** | 8.54 ± 6.13 | 3.41 ± 3.47 |
|  |  |  | **CBCL Other Problems** | 5.03 ± 3.2 | 2.62 ± 2.24 |
|  |  |  | **CBCL Internalization** | 10.82 ± 7.09 | 7.21 ± 4.77 |
|  |  |  | **CBCL Externalization** | 11.8 ± 8.45 | 4.69 ± 4.18 |
|  |  |  | **CBCL Total Problems** | 41.69 ± 24.32 | 20.79 ± 13.07 |
| **Behavior Rating Inventory of Executive Function – 2nd Ed. (BRIEF-2)** | Executive functioning: measures real-world executive skills such as working memory, inhibition, and planning. | | **BRIEF-2 Inhibition** | 14.82 ± 4.27 | 10.38 ± 2.37 |
|  |  |  | **BRIEF-2 Self Monitoring** | 8.67 ± 1.95 | 6.45 ± 1.35 |
|  |  |  | **BRIEF-2 BRI Behavioral Regulation Index** | 23.68 ± 5.43 | 16.83 ± 3.27 |
|  |  |  | **BRIEF-2 Shift** | 13.83 ± 3.07 | 11 ± 2.36 |
|  |  |  | **BRIEF-2 Emotional Regulation** | 14.4 ± 3.87 | 11.31 ± 2.39 |
|  |  |  | **BRIEF-2 ERI Emotional Regulation Index** | 28.23 ± 6.23 | 22.31 ± 4.07 |
|  |  |  | **BRIEF-2 Initiation** | 10.16 ± 2.23 | 7.14 ± 1.75 |
|  |  |  | **BRIEF-2 Working Memory** | 17.21 ± 3.66 | 10.52 ± 2.4 |
|  |  |  | **BRIEF-2 Pianification/Organization** | 16.88 ± 3.46 | 11.34 ± 2.82 |
|  |  |  | **BRIEF-2 Task Monitoring** | 11.59 ± 2.4 | 7.83 ± 2.05 |
|  |  |  | **BRIEF-2 Material Organization** | 11.14 ± 2.56 | 8.1 ± 1.74 |
|  |  |  | **BRIEF-2 CRI Cognitive Regulation Index** | 66.94 ± 11.75 | 44.83 ± 8.46 |
|  |  |  | **BRIEF-2 GEC Global Executive Composite Score** | 118.87 ± 19.86 | 83.97 ± 13.47 |
| **Intolerance of Uncertainty Scale – Parent Version (IUS-P)** | Intolerance of uncertainty: assesses the child’s difficulty tolerating ambiguous or unpredictable situations. | | **Total score** | 30.69 ± 9.57 | 26.38 ± 10.26 |

**Table S3. Description and descriptive statistics for parent-report questionnaires.** Description of parent-report questionnaires used to assess children’s behavior and executive functioning in everyday life. For each scale within the questionnaires (Conners Parent Rating Scale - CPRS, Behavior Rating Inventory of Executive Function - BRIEF-2, Child Behavior Checklist - CBCL, and Intolerance of Uncertainty Scale - Parent version - IUS-P), the table reports the mean ± standard deviation (SD) separately for the ADHD and TD groups.

**Appendix S3. Full statistical models’ details**

Model convergence was checked via R-hat values (max 1.05, [Vehtari et al., 2021](https://www.zotero.org/google-docs/?CUDNi9)), trace plots, and posterior predictive checks. Model performance and influential data points were evaluated using leave-one-out (LOO) cross-validation and k-Pareto diagnostics (‘loo’ package, [Vehtari et al., 2024](https://www.zotero.org/google-docs/?nkzdZE)); observations with Pareto k > 0.7 were flagged.

**S3.1 Model commissions Flanker**

- **Model Family:** Binomial
- **Link Function:** Logit
- **Outcome Variable:** acc (accuracy), with binary trials (1 trial per observation)
- **Fixed Effects:**
- Intercept
- sex

Main effects and all interactions of:

- TrialType
- block
- group
- age
- **Random Effects (by participant id):**
- Random intercept
- Random slopes for TrialType, block and their interaction
- **Dataset:** ‘dat_acc_commission.rds’
- **Observations:** 13,082
- **Sampling Details:**
- 4 MCMC chains
- 4,000 iterations per chain
- 2,000 warm-up iterations
- 2,000 post-warmup samples per chain
- **Total post-warmup draws:** 8,000

| **prior** | **class** | **coef** | **group** |
| --- | --- | --- | --- |
| normal(0, 0.4) | b | age |  |
|  | b | block (50-50) |  |
|  | b | block (50-50 🗙 age |  |
|  | b | block (50-50) 🗙 group (TD) |  |
|  | b | block (50-50) 🗙 group (TD) 🗙 age |  |
|  | b | group (TD) |  |
|  | b | group (TD) 🗙 age |  |
|  | b | sex (M) |  |
|  | b | TrialType (Go invalid) |  |
|  | b | TrialType (Go invalid) 🗙 age |  |
|  | b | TrialType (Go invalid) 🗙 block (50-50) |  |
|  | b | TrialType (Go invalid) 🗙 block (50-50) 🗙 age |  |
|  | b | TrialType (Go invalid) 🗙 block (50-50) 🗙 group (TD) |  |
|  | b | TrialType (Go invalid) 🗙 block (50-50) 🗙 group (TD) 🗙 age |  |
|  | b | TrialType (Go invalid) 🗙 group (TD) |  |
|  | b | TrialType (Go invalid) 🗙 group (TD) 🗙 age |  |
| normal(1.4, 0.3) | Intercept |  |  |
| lkj_corr_cholesky(1) | L |  |  |
|  | L |  | id |
| student_t(3, 0, 2.5) | sd |  |  |
|  | sd |  | id |
|  | sd | block (50-50) | id |
|  | sd | Intercept | id |
|  | sd | TrialType (Go invalid) | id |
|  | sd | TrialType (Go invalid) 🗙 block (50-50) | id |

**Table S4. Prior distribution summary used in the *model_commissions_Flanker*.** The table details prior specifications for each parameter class, coefficient, and group.

**S3.2 Model RTs Flanker**

- **Model Family:** Inverse Gaussian
- **Link Functions:** μ = log; shape = identity
- **Outcome Variable:** RTs (Reaction Times) in seconds
- **Fixed Effects:**
- Intercept
- sex
- preRT (RTs in the preceding trial)
- Acc N-1 (Accuracy in the preceding trial)

Main effects and all interactions of:

- TrialType
- block
- group
- age
- **Random Effects (by participant id):**
- Random intercept
- Random slopes for TrialType, block and their interaction
- **Dataset:** ‘dat_RT.rds’
- **Observations:** 12,431
- **Sampling Details:**
- 4 MCMC chains
- 6,000 iterations per chain
- 3,000 warm-up iterations
- 3,000 post-warmup samples per chain
- **Total post-warmup draws:** 12,000

| **prior** | **class** | **coef** | **group** |
| --- | --- | --- | --- |
| normal(0, 0.05) | b | Acc (n-1) (1) | |
|  | b | age | |
|  | b | block (50-50) |  |
|  | b | block (50-50)🗙 age | |
|  | b | block (50-50)🗙 group (TD) | |
|  | b | block (50-50)🗙 group (TD) 🗙 age | |
|  | b | group (TD) | |
|  | b | group (TD) 🗙 age | |
|  | b | preRT |  |
|  | b | sex (M) |  |
|  | b | TrialType (Go invalid) |  |
|  | b | TrialType (Go invalid)🗙 age | |
|  | b | TrialType (Go invalid)🗙 block (50-50) | |
|  | b | TrialType (Go invalid)🗙 block (50-50)🗙 age | |
|  | b | TrialType (Go invalid)🗙 block (50-50)🗙 group (TD) | |
|  | b | TrialType (Go invalid)🗙 block (50-50)🗙 group (TD) 🗙 age | |
|  | b | TrialType (Go invalid)🗙 group (TD) | |
|  | b | TrialType (Go invalid)🗙 group (TD) 🗙 age | |
| normal(0.01, 0.05) | Intercept |  |  |
| lkj_corr_cholesky(1) | L |  |  |
|  | L |  | id |
| normal(0.1, 0.5) | sd |  |  |
|  | sd |  | id |
|  | sd | block (50-50) | id |
|  | sd | Intercept | id |
|  | sd | TrialType (Go invalid) | id |
|  | sd | TrialType (Go invalid)🗙 block (50-50) | id |
| gamma(0.01, 0.01) | shape |  |  |

**Table S5. Prior distribution summary used in the *model_RT_Flanker*.** The table details prior specifications for each parameter class, coefficient, and group.

**S3.3 Model commissions Addy**

- **Model Family:** Binomial
- **Link Function:** Logit
- **Outcome Variable:** acc (accuracy), with binary trials (1 trial per observation)
- **Fixed Effects:**
- Intercept
- sex

Main effects and all interactions of:

- TrialType
- block
- group
- age
- **Random Effects (by participant id):**
- Random intercept
- Random slopes for TrialType, block and their interaction
- **Dataset:** ‘dat_acc_commission.rds’
- **Observations:** 30,131
- **Sampling Details:**
- 4 MCMC chains
- 4,000 iterations per chain
- 2,000 warm-up iterations
- 2,000 post-warmup samples per chain
- **Total post-warmup draws:** 8,000

| **prior** | **class** | **coef** | **group** |
| --- | --- | --- | --- |
| normal(0, 0.4) | b |  |  |
|  | b | age |  |
|  | b | block (50-50) |  |
|  | b | block (50-50) 🗙 age |  |
|  | b | block (50-50) 🗙 group (TD) |  |
|  | b | block (50-50) 🗙 group (TD) 🗙 age |  |
|  | b | group (TD) |  |
|  | b | group (TD) 🗙age |  |
|  | b | sex (M) |  |
|  | b | TrialType (Go invalid) |  |
|  | b | TrialType (Go invalid) 🗙 age |  |
|  | b | TrialType (Go invalid) 🗙 block (50-50) |  |
|  | b | TrialType (Go invalid) 🗙 block (50-50) 🗙 age |  |
|  | b | TrialType (Go invalid) 🗙 block (50-50) 🗙 group (TD) |  |
|  | b | TrialType (Go invalid) 🗙 block (50-50) 🗙 group (TD) 🗙 age |  |
|  | b | TrialType (Go invalid) 🗙 group (TD) |  |
|  | b | TrialType (Go invalid) 🗙 group (TD) 🗙age |  |
|  | b | TrialType (NoGo) |  |
|  | b | TrialType (NoGo) 🗙age |  |
|  | b | TrialType (NoGo) 🗙 block (50-50) |  |
|  | b | TrialType (NoGo) 🗙 block (50-50) 🗙 age |  |
|  | b | TrialType (NoGo) 🗙 block (50-50) 🗙 group (TD) |  |
|  | b | TrialType (NoGo) 🗙 block (50-50) 🗙 group (TD) 🗙 age |  |
|  | b | TrialType (NoGo) 🗙 group (TD) |  |
|  | b | TrialType (NoGo) 🗙 group (TD) 🗙 age |  |
| normal(1.4, 0.3) | Intercept |  |  |
| lkj_corr_cholesky(1) | L |  |  |
|  | L |  | id |
| student_t(3, 0, 2.5) | sd |  |  |
|  | sd |  | id |
|  | sd | block (50-50) | id |
|  | sd | Intercept | id |
|  | sd | TrialType (Go invalid) | id |
|  | sd | TrialType (Go invalid) 🗙 block (50-50) | id |
|  | sd | TrialType (NoGo) | id |
|  | sd | TrialType (NoGo) 🗙 block (50-50) | id |

**Table S6. Prior distribution summary used in the *model_commissions_Addy*.** The table details prior specifications for each parameter class, coefficient, and group.

**S3.4 Model RTs Addy**

- **Model Family:** Inverse Gaussian
- **Link Functions**:
- mu = log
- shape = identity
- **Outcome Variable:** RTs (Reaction Times) in seconds
- **Fixed Effects:**
- Intercept
- sex
- preRT (RTs in the preceding trial)
- Acc N-1 (Accuracy in the preceding trial)

Main effects and all interactions of:

- TrialType
- block
- group
- age
- **Random Effects (by participant id):**
- Random intercept
- Random slopes for TrialType, block and their interaction
- **Dataset:** ‘dat_RT.rds’
- **Observations:** 24,041
- **Sampling Details:**
- 4 MCMC chains
- 4,000 iterations per chain
- 2,000 warm-up iterations
- 2,000 post-warmup samples per chain
- **Total post-warmup draws:** 8,000

| **prior** | **class** | **coef** | **group** |
| --- | --- | --- | --- |
| normal(0, 0.3) | b | Acc N-1 (0) |  |
| normal(0, 0.3) | b | Age |  |
| normal(0, 0.3) | b | Block (50-50) |  |
| normal(0, 0.1) | b | Block (50-50) 🗙 age |  |
| normal(0, 0.1) | b | Block (50-50) 🗙 group (TD) |  |
| normal(0, 0.1) | b | Block (50-50) 🗙 group (TD) 🗙 age |  |
| normal(0, 0.3) | b | group (TD) |  |
| normal(0, 0.1) | b | group (TD) 🗙 age |  |
| normal(0, 0.3) | b | preRT |  |
| normal(0, 0.3) | b | Sex (M |  |
| normal(0, 0.3) | b | TrialType (Go invalid) |  |
| normal(0, 0.1) | b | TrialType (Go invalid) 🗙 age |  |
| normal(0, 0.1) | b | TrialType (Go invalid) 🗙 block (50-50) |  |
| normal(0, 0.1) | b | TrialType (Go invalid) 🗙 block (50-50) 🗙 age |  |
| normal(0, 0.1) | b | TrialType (Go invalid) 🗙 block (50-50) 🗙 group (TD) |  |
| normal(0, 0.1) | b | TrialType (Go invalid) 🗙 block (50-50) 🗙 group (TD) 🗙 age |  |
| normal(0, 0.1) | b | TrialType (Go invalid) 🗙 group (TD) |  |
| normal(0, 0.1) | b | TrialType (Go invalid) 🗙 group (TD) 🗙 age |  |
| normal(0.01, 0.3) | Intercept |  |  |
| lkj_corr_cholesky(1) | L |  |  |
|  | L |  | id |
| normal(0.1, 0.5) | sd |  |  |
|  | sd |  | id |
|  | sd | Block (50-50) | id |
|  | sd | Intercept | id |
|  | sd | TrialType (Go invalid) | id |
|  | sd | TrialType (Go invalid) 🗙 block (50-50) | id |
| gamma(0.01, 0.01) | shape |  |  |

**Table S7. Prior distribution summary used in the *model_RT_Addy*.** The table details prior specifications for each parameter class, coefficient, and group.

**S3.5 Model quest Addy**

- **Model Family:** Multivariate Gaussian (MV Gaussian for two outcomes)
- **Link Functions**: Identity link for both mean (mu) and standard deviation (sigma) parameters for each outcome
- **Outcome Variable:**
- delta_v (RTs of valid MV block - RTs of valid 50-50 block)
- delta_i (RTs of invalid MV block - RTs of invalid 50-50 block)
- **Fixed Effects:**
- Intercept
- questionnaires composite score
- group
- age
- **Observations:** 120
- **Sampling Details:**
- 4 MCMC chains
- 4,000 iterations per chain
- 2,000 warm-up iterations
- 2,000 post-warmup samples per chain
- **Total post-warmup draws:** 8,000

| **prior** | **class** | **coef** | **group** | **resp** |
| --- | --- | --- | --- | --- |
| normal(0, 0.1) | b |  |  |  |
|  | b |  |  | delta invalid |
|  | b | age |  | delta invalid |
|  | b | group (TD) |  | delta invalid |
|  | b | Q composite |  | delta invalid |
|  | b |  |  | delta valid |
|  | b | age |  | delta valid |
|  | b | group (TD) |  | delta valid |
|  | b | Q composite |  | delta valid |
|  | Intercept |  |  |  |
| normal(0, 1) | Intercept |  |  | delta invalid |
| normal(0, 1) | Intercept |  |  | delta valid |
| lkj_corr_cholesky(1) | Lrescor |  |  |  |
| student_t(3, 0, 2.5) | sigma |  |  | delta invalid |
| student_t(3, 0, 2.5) | sigma |  |  | delta valid |

**Table S8. Prior distribution summary used in the *model_quest_Addy*.** The table details prior specifications for each parameter class, coefficient, and response.

**S3.6 Model NPS Addy**

- **Model Family:** Multivariate Gaussian (MV Gaussian for two outcomes)
- **Link Functions**: Identity link for both mean (mu) and standard deviation (sigma) parameters for each outcome
- **Outcome Variable:**
- delta_v (RTs of valid MV block - RTs of valid 50-50 block)
- delta_i (RTs of invalid MV block - RTs of invalid 50-50 block)
- **Fixed Effects:**
- Intercept
- NPS composite score
- group
- age
- **Observations:** 98
- **Sampling Details:**
- 4 MCMC chains
- 4,000 iterations per chain
- 2,000 warm-up iterations
- 2,000 post-warmup samples per chain
- **Total post-warmup draws:** 8,000

| **prior** | **class** | **coef** | **group** | **resp** |
| --- | --- | --- | --- | --- |
| student_t(3,0,1) | b |  |  |  |
|  | b |  |  | delta invalid |
|  | b | age |  | delta invalid |
|  | b | NPS composite |  | delta invalid |
|  | b |  |  | delta valid |
|  | b | age |  | delta valid |
|  | b | NPS composite |  | delta valid |
|  | Intercept |  |  |  |
| normal(0, 1) | Intercept |  |  | delta invalid |
| normal(0, 1) | Intercept |  |  | delta valid |
| lkj_corr_cholesky(1) | Lrescor |  |  |  |
| cauchy(0,2.0) | sigma |  |  | delta invalid |
| cauchy(0,2.0) | sigma |  |  | delta valid |

**Table S9. Prior distribution summary used in the *model_NPS_Addy*.** The table details prior specifications for each parameter class, coefficient, and response. A lower bound of 0 was applied to the Intercept and standard deviation (sd).

***Appendix S4.* Frequentist statistical analyses results**

**S4.1 Flanker task**

| **Section A: Accuracy** | | | |
| --- | --- | --- | --- |
| **Effect** | **Estimate** | **z-value** | **p-value** |
| **TrialType (I)** | **-0.7** | **-4.7** | **<.001** |
| *block (50-50)* | *0.2* | *0.8* | *.443* |
| **group (TD)** | **0.6** | **3.0** | **.003** |
| **age** | **0.2** | **2.2** | **.030** |
| sex (M) | 0.1 | 0.6 | .558 |
| TrialType (I) x block (50-50) | -0.1 | -0.5 | .626 |
| TrialType (I) x group (TD) | 0.02 | 0.1 | .937 |
| block (50-50) x group (TD) | -0.1 | -0.6 | .563 |
| TrialType (I) x age | -0.1 | -0.9 | .388 |
| block (50-50) x age | 0.1 | 0.5 | .649 |
| group (TD) x age | -0.2 | -0.8 | .406 |
| TrialType (I) x block (50-50) x group (TD) | 0.3 | 1.1 | .292 |
| TrialType (I) x block (50-50) x age | -0.02 | -0.2 | .883 |
| TrialType (I) x group (TD) x age | 0.2 | 1.0 | .335 |
| block (50-50) x group (TD) x age | -0.4 | -1.8 | .072 |
| TrialType (I) x block (50-50) x group (TD) x age | 0.3 | 1.2 | .234 |
| **Section B: Reaction Times** | | | |
| **t-value** | | | |
| **TrialType (I)** | **0.1** | **9.6** | **<.001** |
| **block (50-50)** | **-0.1** | **-4.5** | **<.001** |
| **group (TD)** | **-0.1** | **-2.5** | **.011** |
| **age** | **-0.2** | **-7.8** | **<.001** |
| sex (M) | -0.1 | -1.5 | .146 |
| **preRT** | **0.07** | **13.9** | **<.001** |
| **Acc(n-1) (1)** | **-0.1** | **-5.0** | **<.001** |
| **TrialType(n-1)(incongruent)** | **-0.02** | **-3.2** | **.002** |
| **TrialType (I) x block (50-50)** | **-0.04** | **-2.1** | **.036** |
| TrialType (I) x group (TD) | -0.004 | -0.2 | .852 |
| block (50-50) x group (TD) | 0.01 | 0.5 | .639 |
| TrialType (I) x age | 0.01 | 0.5 | .651 |
| block (50-50) x age | -0.01 | -0.7 | .499 |
| group (TD) x age | 0.02 | 0.6 | .553 |
| TrialType (I) x block (50-50) x group (TD) | -0.004 | -0.2 | .870 |
| TrialType (I) x block (50-50) x age | 0.01 | 0.5 | .586 |
| TrialType (I) x group (TD) x age | -0.02 | -0.7 | .466 |
| block (50-50) x group (TD) x age | 0.02 | 1.1 | .266 |
| TrialType (I) x block (50-50) x group (TD) x age | -0.01 | -0.3 | .791 |

**Table S10. Summary of Frequentist model results for accuracy (Section A) and reaction times (RTs; Section B).** Estimates (log-odds for accuracy and raw values for RTs) are presented along with their associated t- or z-statistic and p-value. Statistically significant results are presented in bold.

***S4.2 Addy task***

| **Section A: Accuracy** | | | |
| --- | --- | --- | --- |
| **Effect** | **Estimate** | **z-value** | **p-value** |
| **TrialType (Invalid)** | **-0.3** | **-2.5** | **.014** |
| **TrialType (NoGo)** | **-0.4** | **-3.7** | **<.001** |
| block (50-50) | -0.1 | -1.4 | .162 |
| **group (TD)** | **0.7** | **6.2** | **<.001** |
| **age** | **0.1** | **2.5** | **.013** |
| **sex (M)** | **-0.2** | **-2.0** | **.046** |
| **TrialType (Go invalid) x block (50-50)** | **0.3** | **2.1** | **.039** |
| TrialType (NoGo) x block (50-50) | 0.1 | 1.2 | .252 |
| ***TrialType (Go invalid) x group (TD)*** | ***-0.4*** | ***-2.1*** | ***.037*** |
| TrialType (NoGo) x group (TD) | -0.2 | -1.0 | .305 |
| block (50-50) x group (TD) | -0.1 | -1.0 | .315 |
| TrialType (Go invalid) x age | -0.01 | -0.1 | .953 |
| TrialType (NoGo) x age | 0.02 | 0.3 | .794 |
| block (50-50) x age | -0.1 | -1.0 | .328 |
| group (TD) x age | 0.1 | 0.9 | .351 |
| TrialType (Go invalid) x block (50-50) x group (TD) | 0.4 | 1.6 | .102 |
| TrialType (NoGo) x block (50-50) x group (TD) | 0.1 | 0.6 | .527 |
| TrialType (Go invalid) x block (50-50) x age | 0.01 | 0.1 | .941 |
| TrialType (NoGo) x block (50-50) x age | -0.1 | -1.2 | .239 |
| TrialType (Go invalid) x group (TD) x age | -0.1 | -0.4 | .725 |
| TrialType (NoGo) x group (TD) x age | -0.2 | -1.1 | .285 |
| block (50-50) x group (TD) x age | -0.2 | -1.2 | .246 |
| TrialType (Go invalid) x block (50-50) x group (TD) x age | 0.2 | 0.8 | .404 |
| TrialType (NoGo) x block (50-50) x group (TD) x age | 0.1 | 0.6 | .561 |
| **Post-hoc contrast: z.ratio** | | | |
| **Block (MV): Go valid - Go_invalid** | **0.5** | **3.9** | **<.001** |
| **Block (MV): Go valid - NoGo** | **0.4** | **4.1** | **<.001** |
| Block (MV): Go invalid - NoGo | -0.1 | -0.4 | .919 |
| Block (50-50): Go valid - Go_invalid | 0.03 | 0.4 | .936 |
| *Block (50-50): Go valid - NoGo* | *0.2* | *2.2* | *.073* |
| Block (50-50): Go invalid - NoGo | 0.2 | 1.9 | .131 |
| Go valid: block (MV) - block (50-50) | 0.2 | 2.0 | .043 |
| **Go invalid: block (MV) - block (50-50)** | **-0.3** | **-2.3** | **.021** |
| NoGo: block (MV) - block (50-50) | -0.04 | -0.3 | .755 |
| ***Go valid: ADHD - TD*** | ***-0.7*** | ***-7.1*** | ***<.001*** |
| ***Go invalid: ADHD - TD*** | ***-0.5*** | ***-3.8*** | ***<.001*** |
| ***NoGo: ADHD - TD*** | ***-0.6*** | ***-4.9*** | ***<.001*** |
| *ADHD: Go valid - Go invalid* | *0.2* | *2.1* | *.088* |
| ***ADHD: Go valid - NoGo*** | ***0.3*** | ***4.0*** | ***<.001*** |
| *ADHD: Go invalid - NoGo* | *0.1* | *1.5* | *.282* |
| ***TD: Go valid - Go invalid*** | ***0.4*** | ***3.2*** | ***.004*** |
| ***TD: Go valid - NoGo*** | ***0.4*** | ***3.3*** | ***.003*** |
| *TD: Go invalid - NoGo* | *0.02* | *0.1* | *.992* |
| **Section B: Reaction Times** | | | |
| **t-value** | | | |
| **TrialType (Go invalid)** | **0.1** | **4.0** | **<.001** |
| **block (50-50)** | **0.1** | **4.4** | **<.001** |
| group (TD) | 0.1 | 1.5 | .143 |
| **age** | **-0.1** | **-3.1** | **.002** |
| sex (M) | -0.03 | -0.7 | .490 |
| **preRT** | **0.13** | **28.9** | **<.001** |
| **Acc(n-1) (0)** | **0.1** | **11.4** | **<.001** |
| **TrialType(n-1)(invalid)** | **-0.01** | **-2.3** | **.020** |
| **TrialType(n-1)(noGo)** | **-0.26** | **-23.1** | **<.001** |
| **TrialType (Go invalid) x block (50-50)** | **-0.1** | **-3.9** | **<.001** |
| TrialType (Go invalid) x group (TD) | 0.04 | 1.4 | .159 |
| **block (50-50) x group (TD)** | **-0.1** | **-3.9** | **<.001** |
| TrialType (Go invalid) x age | -0.003 | 0.2 | .849 |
| block (50-50) x age | -0.01 | -0.7 | .500 |
| group (TD) x age | 0.1 | 1.5 | .127 |
| TrialType (Go invalid) x block (50-50) x group (TD) | -0.03 | -1.2 | .246 |
| TrialType (Go invalid) x block (50-50) x age | 0.005 | 0.3 | .758 |
| TrialType (Go invalid) x group (TD) x age | -0.04 | -1.4 | .154 |
| block (50-50) x group (TD) x age | -0.02 | -0.8 | .415 |
| TrialType (Go invalid) x block (50-50) x group (TD) x age | 0.01 | 0.5 | .625 |
| **Post-hoc contrast: z.ratio** | | | |
| **Block MV: Go valid – Go invalid** | **-0.1** | **-7.8** | **<.001** |
| **Block 50-50: Go valid – Go invalid** | **-0.03** | **-3.5** | **.001** |
| Go valid: block (MV) - block (50-50) | -0.01 | -0.6 | .578 |
| **Go invalid: block (MV) - block (50-50)** | **0.058** | **4.1** | **<.001** |
| **ADHD: block (MV) - block (50-50)** | **-0.03** | **-2.1** | **.037** |
| **TD: block (MV) - block (50-50)** | **0.1** | **4.6** | **<.001** |
| **Block MV: ADHD - TD** | **-0.08** | **-2.0** | **.047** |
| Block 50-50: ADHD - TD | 0.03 | 0.8 | .455 |

**Table S11. Summary of Frequentist model results for accuracy (Section A) and reaction times (RTs; Section B).** Estimates (log-odds for accuracy and raw values for RTs) are presented along with their associated t- or z-statistic and p-value. Italicized results indicate differences compared to those reported in the paper using the Bayesian approach. Statistically significant results are presented in bold.

***S4.3 Addy task & Questionnaire composite score***

| **Response** | **Term** | **Estimate** | **t-value** | **p-value** |
| --- | --- | --- | --- | --- |
| **Delta valid** | Q composite | 0.1 | -1.4 | .156 |
|  | **group (TD)** | **0.6** | **0.6** | **.011** |
|  | age | 0.1 | 1.3 | .208 |
| **Delta invalid** | Q composite | -0.1 | -1.3 | .250 |
|  | **group (TD)** | **0.2** | **1.2** | **.025** |
|  | age | 0.04 | 0.5 | .656 |

**Table S12. Summary of Frequentist model results for Addy’s Delta scores and questionnaires composite score.** Summary of linear regression results examining the relationship between broader behavior (questionnaires’ composite score) and CC adaptation (Delta Valid and Delta Invalid). Estimates, t-values, and p-values are reported. Statistically significant results are presented in bold.

***S4.4 Addy task & NPS composite score in ADHD***

| **Response** | **Term** | **Estimate** | **t-value** | **p-value** |
| --- | --- | --- | --- | --- |
| Delta valid | NPS composite | 0.3 | 1.8 | .077 |
|  | Age | -0.04 | -0.4 | .726 |
| **Delta invalid** | **NPS composite** | **0.4** | **2.4** | **0.20** |
|  | Age | -0.04 | -0.4 | .726 |

**Table S13. Summary of Frequentist model results for Addy’s Delta scores and NPS composite score in ADHD.** Summary of linear regression results examining the relationship between neuropsychological functioning (NPS composite score) and CC adaptation (Delta Valid and Delta Invalid). Estimates, t-values, and p-values are reported. Statistically significant results are presented in bold.

**Figure S3. Delta Valid and Delta Invalid and Group.** The plot displays scaled scores. Panel A. On the y-axis the Delta Valid (i.e., mean RT of valid trials in Block MC - Block 50:50), and on the x-axis the Groups (i.e., ADHD, TD). Boxplots indicate the median (horizontal line), interquartile range (box), and the full data range (whiskers); individual data points are overlaid. The blue color-scale is graded as a function of age in months, with lighter colors for older children and darker colors for younger children. Panel B. Same layout as Panel A, but showing Delta Invalid scores instead.
